# Behavioral science of voting in hypothetical utopia and dystopia scenarios: a predictive modeling approach

**DOI:** 10.3389/fpsyg.2025.1713314

**Published:** 2026-01-12

**Authors:** Janne Kauttonen, Jyrki Suomala

**Affiliations:** 1 Digital Transition and AI, Haaga-Helia University of Applied Sciences, Helsinki, Finland; 2 Neurolab, Laurea University of Applied Sciences, Espoo, Finland

**Keywords:** contextual framing, partisanship, political psychology, predictive modeling, scenario analysis, voting behavior

## Abstract

We investigated how near-future utopia and dystopia scenarios affect electorates' valuation of the leading Finnish politicians in the multiparty democracy. Using an online questionnaire with 1,109 respondents, we collected valuations across six attributes (e.g., trustworthiness, suitability as prime minister) both at baseline and after random assignment to a scenario. Valuations were first made for the present day and second in a near-future scenario. The strongest predictors for the electorates' valuations were the party, gender, and familiarity of the candidate, as well as liberal or conservative attitudes of the voter. Conversely, the effect of the scenarios was found to be weaker, and both scenarios tended to reduce the valuations of politicians. Devoted voters were less likely to change their opinion about their representative than non-devoted voters. The only exception to this was a notable increase in valuation of the right-wing politician in the dystopian scenario. In addition, we found that liberality was associated with higher sensitivity to opinion change among women. This study reveals the interplay between voter-, politician-, and context-related factors in political judgment. It also demonstrates the value of combining predictive machine learning with inferential statistical modeling to analyze complex behavioral data.

## 1 Introduction

Voting is a complex decision process shaped by multiple interacting factors. Prior research shows that electorate-related factors such as ideology, personality, and demographic background influence political evaluations (Kauttonen and Suomala, 2019; Lau et al., 2018). At the same time, politician-related factors like competence, trustworthiness, gender, familiarity, and leadership style, also play a central role in how voters form judgements about political actors (Ditonto, 2017; Garzia, 2011). Together, these electorate- and politician-related factors represent two well-established pillars of the electoral decision-making literature (Kim et al., 2010). In the present study, we examine voting decisions focusing on how alternative narrative contexts, specifically utopian and dystopian future scenarios, shape evaluations of Finnish political leaders.

The situational context in which evaluations are made can shape how citizens interpret political information and perceive political leaders. Although political science has made substantial progress in explaining voter choice through sociological, economic, and psychological determinants, considerably less is known about how situational factors influence the voting decision process (Blais and St-Vincent, 2011). According to the situational perspective, political judgments are highly responsive to contemporary conditions and evolving features of the political environment (Kim et al., 2010; McCann et al., 1994; Shiller, 2019). These contextual influences may interact with voter and candidate characteristics, adding further complexity to the formation of electoral preferences.

One situational factor that remains understudied in election research is the role of narratives, which are the coherent storylines through which individuals interpret political issues, events, and possible futures (Groth, 2019). Narratives help people organize information, assign meaning, and anticipate consequences by embedding political developments in broader causal and moral structures (Dunbar, 2004; Shiller, 2019). They can evoke emotions, highlight risks or opportunities, and activate expectations about political leadership before specific candidates are even evaluated. Despite the centrality of narratives in human cognition, their function in shaping voter evaluations has received limited empirical attention.

Next, we review prior research on situational and narrative influences, electorate and politician-related factors and modeling of voter behavior.

### 1.1 Narratives as part of situation-related factors

Prior research on situational factors has shown that voting in a school can increase support for education initiatives, whereas voting in a church can increase conservative preferences (Berger et al., 2008; Rutchick, 2010). Other work demonstrates that exposure to mortality cues, such as images of cemeteries or reminders 9/11, as well as incidental stimuli such as unpleasant odors, messy rooms, or uncomfortable seating, can shift political attitudes, often toward more conservative positions (Hibbing et al., 2014; Landau et al., 2004). These findings highlight that concrete situational cues can shape voters' emotions, attentional focus, and evaluations, even outside conscious awareness.

These studies, however, primarily focus on physical or sensory aspects of situations. Much less attention has been paid to how interpretive contexts shape political judgment. It is therefore essential to examine situational influence at a broader and more interpretative level. In this study, we adopt that perspective and focus on narrative contexts as a critical yet understudied dimension of political evaluation.

In psychology, frames refer to cognitive structures that organize experience and guide evaluation (Goffman, 1974). Classic work in decision-making demonstrates that alternative framings of identical information can systematically alter preferences (Tversky and Kahneman, 1981; Kahneman and Tversky, 1984). In political communication, framing involves selecting and emphasizing aspects of reality in ways that shape causal attributions, moral evaluations, and policy preferences (Entman, 1993; Chong and Druckman, 2007). These frameworks highlight that situational context is not only external and physical but also mental and interpretive.

Building on this, recent work emphasizes narratives as a particularly powerful form of framing. Narrative frames are coherent storylines that structure events into temporal sequences, assign roles such as victims or leaders, and embed implicit assumptions about causes, responsibilities, and outcomes (Bandelow et al., 2025). Psychological research on narrative transportation shows that becoming immersed in a narrative increases story-consistent beliefs and decreases counterarguing (Green and Brock, 2000; Green and Appel, 2024). Thus, narratives influence evaluations by shaping how individuals imagine political situations and possible futures.

However, evaluations of political leaders never occur in a vacuum. They are embedded in broader interpretive contexts, often narrative in nature, that activate expectations about the state of society. Utopian and dystopian narratives, in particular, have been shown to evoke contrasting emotional and cognitive orientations, with utopian narratives highlighting stability, prosperity, and collective agency, while dystopian narratives emphasize threat, crisis, and loss of control (Nilsson et al., 2019; Harrigan et al., 2020; Holman et al., 2022). Experimental evidence further suggests that dystopian scenarios can heighten perceptions of crisis and strengthen support for decisive political leadership (Holman et al., 2022). In contrast, utopian scenarios can foster openness, optimism, and trust in collective solutions (Cools et al., 2024). Even when fictional, such narratives could evoke psychologically realistic responses (Green and Appel, 2024) and can shift attitudes, trust, and perceived leadership needs. For this reason, narrative scenarios can be conceptualized as situational frames that alter which leader qualities become more salient, leading to shifts in how political leaders are evaluated when the surrounding narrative contexts change.

Our study places narrative framing between utopian and dystopian near-future scenarios at the center of analysis. Rather than treating situational context as a background element, we examine how alternative narrative frames shape perceptions of key political leaders in Finland.

### 1.2 Electorate-related factors

Voting behavior is influenced by a variety of electorate-related factors, including voter demographics, psychological traits, and socioeconomic status. Previous studies have found that there are relationships between the personality—measured with the Big-Five framework—of voters and their political orientations. Political conservatism had a positive correlation with Conscientiousness and a negative correlation with Openness to Experience (Carney et al., 2008; Sibley et al., 2012). More broadly, conservatives are more orderly and better organized, whereas liberals are more open-minded and curious (Carney et al., 2008). In addition, conservatives tend to pay more attention to the unpleasant things in the environment, and they pursue a more risk-avoidant strategy compared to liberals (Hibbing et al., 2014; Shook and Fazio, 2009). Most of the studies about the relationship between the electorate's personality and political orientation have been researched in two-party democracies like the USA and the question is, do the multiparty democracies have so a clear effect among voters.

A previous study found that the conservativeness, age, gender, ordinality, and social activity of the voter had a causal connection with the valuation level of a prime minister candidate (Kauttonen and Suomala, 2019). In addition, conservativeness, age, and gender had direct connections also with trustworthiness and familiarity of politicians. A study by Lau et al. (2018) reported that older people avoid cognitively demanding political decisions. Instead, they gravitate much more to Fast and Frugal decision-making. This result is consistent with the findings (Strømsøa et al., 2011) that older people have a general tendency to make decisions by memory when rejecting new information related to situations.

Whereas the voting rates of females are usually higher than males, the relationship between age and participation is non-linear, and it varies substantially by other demographic variables (Ansolabehere and Hersh, 2013). An interesting question is what the relationship between gender and other demographic variables is to the evaluation of politicians overall.

Furthermore, voters tend to make motivated reasoning when choosing a candidate (Kunda, 1990; Lau et al., 2018). In this case, a voter judges a politician according to how much the views of the politician are parallel to the voter's personal views. Marks et al. (2019) found a phenomenon called “spillover”, according to which an individual trusts the like-minded person they appreciate, regardless of their competence or behavior. Motivated reasoning has been found especially among European electorates who have strong partisanship levels (Huddy et al., 2018). Also, in Finland and the USA, party affiliation of the candidate was found to be the most critical characteristic among citizens (Badas and Stauffer, 2019; Coffé and von Schoultz, 2021).

### 1.3 Politician-related factors

In addition to electorate characteristics, political candidates‘ personal attributes, backgrounds, and behaviors significantly influence voter preferences. In most democracies, leaders of their parties are an influential electoral force in election campaigns, and they are often also prime minister candidates (Bean and Mughan, 1989). There is growing evidence that voters care more about politicians' personal characteristics than other politically relevant information like policy stances (Ditonto, 2017; Garzia, 2011; Holian and Prysby, 2014; Wattenberg, 1991).

Much evidence suggests that the gender of politicians is subject to stereotypes based on personality traits, issue competencies, and ideology (Ditonto, 2017). Female candidates can be seen as more gentle, warm, emotional, trustworthy and honest than male candidates. Moreover, voters often assume that women have proficiency in “compassion issues” like education, healthcare, and childcare, whereas issues like crime, the military, and the economy are seen as the arena of male politicians. In addition, information related to incompetence affects voter evaluation of women's policies, but not the evaluation of male politicians (Ditonto, 2017).

Holli and Wass (2010) found that men were more prone than women to choose a candidate of their own gender. They concluded that in Finland, gender-based voting affects electoral outcomes regardless of the type of election. The same conclusions were also drawn by another study in Finland (Giger et al., 2014) and USA (Brians, 2005). Particularly in nonpartisan elections, gender affinity can meaningfully shape electoral outcomes (Badas and Stauffer, 2019). While extensive research from the United States has explored the role of gender-based stereotypes, it is important to test whether these findings generalize to different political systems, such as the multi-party democracy in Finland. Thus, the role of gender-based stereotypes in the actual voting process is unclear.

Party leaders and ministers are most visible in the media, and they are often very familiar to voters. It has been found that enhancing the familiarity of a candidate increases positive attitudes of the candidate among voters (Esterling et al., 2015). Thus, familiarity is probably one of the key factors influencing the voting decision.

Previous studies have found that perceptions of a politician's competence is one of the critical factors when voters evaluate politicians (Ditonto, 2017; Holian and Prysby, 2014; Kauttonen and Suomala, 2019). In addition, communication and collaboration skills, and trustworthiness are important attributes for a political leader (Barisione, 2009; Kauttonen and Suomala, 2019). Moreover, according to voters, a political leader should be one of them, and they should advocate for the benefits of the voters (Garzia, 2011). Additionally, a political leader should be fair, if necessary, tough, and capable of making difficult decisions (Bean and Mughan, 1989; Rule et al., 2010).

Also, the voting system itself affects the choice. Finland has a multi-party system where voters vote directly for the person they want to see elected. Elections are proportional following a d‘Hondt system of the party list (von Schoultz, 2017). Neither is the electorate given the option to vote for a party *per se*. As the d'Hondt formula of allocating seats favors large parties, in Finland small parties usually take the opportunity of joining an electoral alliance with one or more parties (von Schoultz, 2017). As a result, the importance of an individual politician is reduced while the selection of the party is increased.

In addition, Finland is a parliamentary multi-party democracy characterized by high institutional trust, consensus-oriented policymaking, and broad political participation typical of Nordic welfare states (Lægreid and Rykkja, 2020). Executive power is divided between a Parliament-elected Prime Minister, who directs domestic and EU policy, and a directly elected President with more limited responsibilities, mainly in foreign and security affairs. Finland granted universal suffrage in 1906, becoming the first country in the world where both women and men received full voting rights and eligibility for office. Finland has also had a woman President (2000–2012), and in 2019 the country formed a coalition government led by Prime Minister Sanna Marin, in which all governing parties were chaired by women, reflecting the long-standing trend of strong female political representation in Finland (Helimäki et al., 2024). In early 2020, when our data were collected for this study, Finland's population was slightly female-majority (approx. 50.4% women, 49.6% men), and the period overlapped with the onset of the COVID-19 pandemic. The first nationwide restrictions began in mid-March 2020, whereas the early phase of our data collection (January–February) preceded these measures.

### 1.4 Predicting voter behavior

Explaining and predicting voter behavior are essential yet notably difficult goals in political science (Cranmer and Desmarais, 2017; Wattenberg, 1991). Controversies about the level of predictability of behavioral research in general and political science, especially, analyzed using traditional statistical inference have sparked interest in more efficient techniques for analyzing the results of behavioral studies (Holian and Prysby, 2014; Yarkoni, 2022). While traditional statistical methods like linear regression are common, there is growing interest in ML to capture complex, non-linear relationships that these models may miss (Holian and Prysby, 2014; Montgomery and Olivella, 2018). Traditional linear models typically assume that factors influence the outcome in a constant, additive way (fitting “straight lines” to data). In contrast, ML algorithms learn flexible decision rules that can detect “tipping points” and conditional patterns. For instance, a ML model could reveal that ideology might only predict a choice when combined with specific demographic traits without the researcher needing to specify these interactions in advance. This makes them well suited for uncovering complex patterns in voters' evaluations (Cranmer and Desmarais, 2017; Montgomery and Olivella, 2018; Yarkoni, 2022).

ML can be useful for theory building because it can uncover relationships between variables that might not have been obvious when using linear statistical analysis, and can, in this way, suggest features thereof that should be excluded from the statistical models (Cranmer and Desmarais, 2017; Johnson et al., 2019; Orrù et al., 2020). Predictive models have been successfully applied to anticipate voting preferences from social media data (Kristensen et al., 2017), forecast Brexit voting patterns (Becker et al., 2017), and estimate swing voter likelihood with high precision (Hare and Kutsuris, 2023). A primary benefit of ML is its capacity to maximize predictive power with new data (Montgomery and Olivella, 2018). Although the intricacy of these models can create “black box” challenges, methods such as Shapley additive explanations (SHAP; Lundberg and Lee, 2017) can still provide model-agnostic interpretations. This allows researchers to assess the influence of individual variables and their interactions, thereby using ML's predictive accuracy to enhance theory building and testing (Orrù et al., 2020). Further discussion on this topic is available in online Supplementary Material 2.

### 1.5 Current study

In the present study, we examine voting decisions from an integrated perspective that includes situational narratives, electorate-related factors, and politician-related factors. Based on the situational framing literature, we hypothesized that exposure to strong, polarized narrative manipulations (utopia and dystopia) would elicit measurable shifts in voter opinion compared to the baseline. Furthermore, we anticipated that these shifts would not be uniform but would interact with electorate-related factors, specifically partisanship and personality traits. Focusing on Finnish political leaders, we combined narrative manipulations with extensive data on voters and politicians as well as modern machine learning and statistical modeling techniques.

We collected survey data from 1109 Finnish adults evaluating 9 real Finnish politicians. We then applied two regression approaches to analyze our multidimensional data: gradient boosted tree regression and Bayesian mixed-effects ordinal linear regression. While the former allowed us to leverage ML in capturing nonlinear relationships and variable interactions, the latter allowed us to take into account hierarchical structure in our data with robust Bayesian statistical machinery. These complementary methods allowed us to gain more comprehensive insight into the factors affecting respondents' decisions under narrative manipulation.

## 2 Data and methods

### 2.1 Participants

Participants were recruited via mass emails sent to students and staff at four Finnish higher education institutions, as well as through social media channels. A total of 1,109 complete responses were received from adult participants (655 women). Completing the full questionnaire allowed participants to enter a lottery for 20 gift cards (each worth 25 euros).

### 2.2 Utopian and dystopian narratives

Humans tend to organize their thinking through narratives about possible futures (Geary, 2005). Following prior research on utopian and dystopian framing (see Introduction), we used two contrasting narrative scenarios to examine how imagined futures shape political evaluations. Participants were asked to imagine Finland 5 years in the future (i.e., 2025) under two alternative conditions—one utopian and one dystopian. The essential question was how situational framing affects respondents' valuation decisions. The narratives were as follows (translated from Finnish):

*Utopia*: *The Finnish economy is doing well, and you have plenty of excess cash after necessary living expenses. You have not resorted to any income support. You rarely need a doctor and a visit to the doctor is free. Your workplace makes a lot of use of artificial intelligence and there is little routine work left. Your working time is 6.5 h a day with plenty of holidays and free time. Unemployment in Finland is at a record low. Basic livelihood is good also for those without work and allows them to study, have hobbies, take care of children or spend high-quality free time. You live in a house and drive cars which have very low emissions thanks to advanced technology. Climate change is well under control. Food is cheap and produced sustainably. As a result of high international stability, the number of refugees is low and most of them integrate well into society. You and the other Finns are well and the future looks positive*.

*Dystopia: The Finnish economy is doing poorly and your salary goes fully to necessary living expenses. You have often resorted to income support. You need a doctor often and a visit to the doctor costs a lot. Your workplace does not utilize artificial intelligence at all and there exists lots of routine work. You work 9.5 h a day and have little vacation and free time. Unemployment in Finland is at a record high. Basic income is low for those without work and does not allow them to study, have hobbies, take care of children or spend high-quality free time. You live in a house and drive cars which have very high emissions because of undeveloped technology. Climate change is not under control. Food is expensive and produced unsustainably. As a result of international instability, the number of refugees has increased and most of them are being marginalized. You and other Finns feel hopeless and the outlook for the future is negative*.

Each subject was randomly assigned to one of these scenarios.

While much of the existing research focuses on subtle environmental primes, our study employs starkly contrasting utopian and dystopian narratives. This choice is deliberate for three key reasons. First, such powerful, emotionally resonant scenarios are designed to elicit stronger and more measurable shifts in valuation than subtle cues, allowing for a more robust test of the stability of political attitudes. Second, these frames reflect the polarized nature of modern political discourse, where politicians often frame the future in terms of either societal flourishing (a utopia) or collapse (a dystopia) to mobilize support. Finally, this design allows us to test specific, theoretically grounded hypotheses about voter behavior under perceived conditions of extreme stability vs. crisis. Employing polarized narratives provides a clear experimental manipulation to assess how imagined futures can reframe the electorate's evaluation of current political figures.

### 2.3 Constructing the online questionnaire

Our online questionnaire contained five segments in the following order: (A) personal information of the responder, (B) first valuation of each politician, (C) voting behavior of responders, (D) scenario (utopia or dystopia with equal chance), and (E) second valuation of each politician. The scenario was considered as a treatment effect for the second valuation. Since background information (personality and voting behavior) was constant for both valuation rounds, each respondent was used as her/his own control when studying the effect of the scenario. The design and implementation were similar to those used in a previous study (Kauttonen and Suomala, 2019). The research protocol was approved by the appropriate review board at Laurea University of Applied Sciences. All participants were informed about the study's purpose on the introductory page of the online survey. Participation was fully voluntary. The data were collected and analyzed anonymously.

We chose nine politicians, one from each political party or group in Finland having a seat in the Finnish parliament in 2020. Together, the parties had 97.9% support in the parliamentary elections of 2019. According to a recent poll using the classical “left-right” categorization (Puoluekartta: Oikeistossa kuusi, keskusta-oikeistossa kaksi ja vasemmistossa kolme puoluetta, 2019), the nine parties received scores between 1.3 and 4.3 on the scale of 1–5 (1 = full left-wing, 5 = full right-wing). In January-February 2020, the support of the nine parties ranged between 1.3% and 23.3% (error margin 2.0%; Ylen kannatusmittaus: Keskustan kannatus sukeltaa jälleen, vihreiden suosio kasvaa ja perussuomalaisten kannatushuippu takana, 2020). However, we note that our evaluations were based on individual politicians, not parties as a whole. This is justified because the party leaders are almost without exception prime minister candidates, and this has an influential electoral force in election campaigns (Bean and Mughan, 1989; Garzia, 2011; Wattenberg, 1991). The politicians, their abbreviations, gender, age and party (with the above statistics) are listed in Table 1. From now on, we use these abbreviations (i.e., JH, LA, SM, MO, HH, AH, PO, KK, SE) to simplify the notation.

**Table 1:** The nine politicians in the study.

**Politician (abbreviation)**	**Gender**	**Age^a^**	**Party**	**Party political stance^b^ [1–5]**	**Party support^c^%**
Jussi Halla-Aho (JH)	Male	48	Perussuomalaiset (Finn party)	3.8	23.3
Li Andersson (LA)	Female	32	Vasemmistoliitto (Left alliance)	1.3	8.3
Sanna Marin (SM)	Female	34	SDP (Social democratic party)	2.2	16
Mari Ohisalo (MO)	Female	34	Vihreä Liitto (Green League)	2.4	13.3
Harry Harkimo (HH)	Male	66	Liike Nyt (Movement now)	4.1	1.3
Anna-Maja Henriksson (AH)	Female	56	Ruotsalainen Kansanpuolue (Swedish people's party)	3.8	4.4
Petteri Orpo (PO)	Male	50	Kokoomus (National coalition party)	4.3	17.5
Katri Kulmuni (KK)	Female	32	Keskusta (Center party)	3.5	10.8
Sari Essayah (SE)	Female	52	Kristillisdemokraatit (Christian democrats)	3.7	3.0

^a^January 2020.

^b^Poll 1.-6.2.2019.

^c^Poll 13.1-4.2.2020.

Abbreviations are shown in parentheses. The table also contains the stance and support of the parties they represent. The stance measure ranges from 1 (full “left-wing”) to 5 (full “right-wing”).

Responder background information included gender (male/female/no response), age, and 8 personal attributes. The choice of these attributes was guided by prior research linking personality and values to political behavior. The latter included claims of type “I am…” followed by disciplined, carefree, open to new experiences, conventional, conservative and socially liberal. Specifically, the disciplined/carefree and open/conventional items were selected as proxies for the Big Five dimensions of Conscientiousness and Openness to Experience, respectively, which are established correlates of political orientation. The liberal/conservative self-assessment directly captures the ideological leaning of the voter. Furthermore, the question on defending an unpopular belief was included to gauge outspokenness, while the question on speculative investments serves as a behavioral proxy for risk tolerance, a fundamental trait in decision-making under uncertainty. These questions were based on the Big Five personality scale (McCrae and John, 1992) and risk tolerance scale (Weber et al., 2002). Each politician was evaluated via questions of suitability as a prime minister, trustworthiness, cooperation skill, righteousness, patriotism and decision-making skill. The exact questions are listed in Table 2 with a copy of the full survey (in Finnish) found in the online Supplementary Material 1.

**Table 2:** Variables, values, types and classes in the data.

**Variable**	**Description or question (values)**
**Predictors**	
gender	What is your gender? (male, female)
match_gender	Does gender of responder and politician match? (yes/no)
match_party	Does the party of the politician match the last and/or upcoming vote (no, last, next, both)
age	What is your age group? (18…60+)
familiarity	How familiar is this politician to you? (0…6)
discipline	I am disciplined/carefree (0…13)
liberality	I am liberal/conservative (0…13)
openness	I am open to new experiences/ordinary (0…13)
outspokenness	I defend an unpopular belief I hold in a social situation (0…6)
investor	I invest 5% of my annual income in highly speculative stocks (0…6)
scenario	Response scenario (baseline, dystopia, utopia)
politician	Politician (9 options)
**Responses**	
Suitability	He/she is suitable as a Prime Minister of Finland (0…6)
Trustworthiness	He/she is trustworthy (0…6)
Cooperationskill	He/she is capable of collaboration (0…6)
Righteousness	He/she is righteous (0…6)
Patriotism	He/she acts best for Finland (0…6)
Decisionmaking	He/she is capable of making decisions (0…6)

Multivariate models fit into the data with 13 predictor variables and 6 outputs.

### 2.4 Data preprocessing

First, the raw questionnaire response data were converted into a table with each row representing one subject and all rows with incomplete data were removed. We excluded subjects whose scenario reading time was less than 15 s, which indicated that these subjects were mostly motivated by the lottery. After these steps, our dataset included *N* = 1,109 subjects, which were randomly assigned into a training set (*n* = 998 with 589 females) and a testing set (*n* = 111 with 66 females).

The dataset contained a total of 19 variables of interest, from which 13 were predictors (inputs) and six dependent responses (outputs). All variables are listed in Table 2. Note that match_gender and match_party were derived variables computed from the raw responses. The categorical predictor variables (total of five) were one-hot encoded before entering CatBoost regression. The three personality trait variables, “liberality”, “discipline” and “openness” were each created from two variables by combining two seven-item Likert-scale variables, hence they had 14 unique levels compared to others with only seven levels. Age and personality characteristics predictors were considered as numerical covariates. Response variables were considered numerical (in gradient boosting) or ordinal (in Bayesian regression), for reasons discussed later for regression analysis.

### 2.5 Regression analysis via gradient boosted trees

We applied the CatBoost library (Prokhorenkova et al., 2018), https://github.com/catboost (version 1.0.6). Gradient boosted trees were chosen for their good predictive accuracy and ability to capture complex, non-linear relationships and high-order interactions directly from the data without strong *a priori* assumptions. Tree-based models are highly flexible and excel at identifying the most influential predictors in a data-driven manner. Our primary motivation for this method was to maximize predictive power and uncover potentially unexpected patterns in how voters evaluate politicians. Tree-based methods are particularly valuable in settings where one wishes to make accurate predictions in the context of a generally unknown data-generating process with potential nonlinearities, interactions, and many (potentially irrelevant) covariates (Montgomery and Olivella, 2018).

To make predictions with ordinal response variables with CatBoost, we assumed that the output values were on an equidistant, continuous scale. After one-hot encoding, the total number of predictor variables entered in CatBoost was 22. For interpretations and model analysis, we used the SHAP method (Lundberg and Lee, 2017) that applies information theory to pinpoint the impact of each predictor on the outcomes. It is a method for interpreting machine learning models by calculating SHAP values, which quantify the contribution of each feature to the model's predictions, such as tree-based gradient boosting methods, including CatBoost. Based on game theory, the SHAP algorithm assigns each feature a contribution score that indicates how much it influences a prediction (Lundberg et al., 2020). We used stochastic grid-search and five-fold cross-validation (80% for training, 20% for testing) to optimize hyperparameters for the training data. The final model performance was tested with separate test data.

After training the model, we computed SHAP values and used permutation to check whether SHAP values were on average non-zero. By assuming independence between subjects, we applied permutation tests to one-sample (difference of mean against zero), two-sample (difference of two independent group means against zero) and Spearman's rank (difference of correlation coefficient against mean) implemented in the Python package *permute* (https://github.com/statlab/permute). Corresponding *P*-values were adjusted with the False Discovery Rate (FDR) method (Benjamini and Hochberg, 1995). Because of our assumption of response equidistance, SHAP values represent steps in the Likert scale, i.e., SHAP value 1.0 corresponds to one step and 0.5 half-step and so on. Performance of the model was assessed using Pearson's R^2^ for the separate test data.

### 2.6 Regression analysis via Bayesian linear mixed-effects ordinal regression

We used the *R* package *brms* (Bürkner, 2021), https://github.com/paul-buerkner/brms (version 2.19.0) to fit models and *emmeans* package, https://github.com/rvlenth/emmeans (version 1.8.4), to compute *post-hoc* tests. The Bayesian mixed-effects ordinal regression model was employed for its strength in statistical inference. While not maximizing predictive power, the main advantage of the Bayesian framework is its ability to quantify uncertainty for every parameter through posterior distributions and Highest Density Intervals (HDIs), which provides a more complete and intuitive interpretation than traditional null-hypothesis testing. We fitted a single common model that included all politicians. The model also included politicians as random effects and the correlated intercepts with subjects. The rationale is that this model allowed us to make inferences about the larger population of politicians, not only the ones included in this study.

For the common model, we fitted the following equation:


$$\begin{array}{l} \text{y}\sim \text{1}+\text{scenario}:(1+\text{age}+\text{gender}+{\text{match}}_{\text{gender}}+\text{familiarity}\\ \;+{\text{match}}_{\text{party}}+{\text{gender:match}}_{\text{gender}}+\text{TRAITS})\\ \;+\text{scenario:}\left({\text{match}}_{\text{party}}+{\text{match}}_{\text{gender}}+\text{gender}\right):\\ \;\left(\text{TRAITS}+\text{familiarity}\right)+\;\left(\text{1}|\text{subject}\right)\\ \;+\;\left(\text{1}|\text{candidate}\right)+\left(\text{1}|\text{candidate:subject}\right), \end{array}$$ (1)

where

TRAITS := discipline + liberality + openness + outspokenness + investor

Here, *y* is one of the six responses on a continuous (latent) scale, which was converted to an ordinal response (Bürkner and Vuorre, 2019). Interactions up to three-way were included (such as “scenario:gender:familiarity”), with one of the variables always being “scenario” since other predictors were independent of it. The formula was the same for the individual, single-politician models, but without *match*_*gender*_ term and two last random-effects terms. The latent scale was then converted into an ordinal response via cumulative distribution with a logistic link function. Because of the large model and sample size, we considered ordinal numerical predictors as scale variables (i.e., equidistance). Before training, all covariates were normalized to have zero mean and a standard deviation of one. The total number of fixed-effects (population) coefficients was 182 for each dimension, i.e., a total of 1,092. Additional information of fitting can be found in the online Supplementary material.

In order to make statistical interpretations, we computed Highest Density Intervals (HDI) from the models. For this, we applied *emmeans*, which compute marginal means and linear trends related to factorial and numerical predictors starting from posterior distributions. We applied thresholds 95%, 99%, and 99.9% when reporting the HDI results. We also used *emmeans* to compute related contrasts, both first (“difference of means”) and second difference (“difference in differences of means”; DID). DID is a quasi-experimental design that estimates causal effects by comparing the changes in outcomes between a treatment group (the baseline) and a control group (scenario). By differencing twice, we mitigate the potential bias from group differences and from period effects unrelated to the intervention (see, e.g., Lechner, 2010).

The magnitude of contrast values represents effects on the latent log-odds (logit) scale. Exponentiating these values yields the corresponding odds ratios. To convert between log-odds ratios (OR) and standardized mean differences (Cohen's *d*) (Cohen, 1988), one may use the logistic distribution's standard deviation ($\sigma =\frac{\pi }{\surd 3}\approx 1.8138$; Chinn, 2000) and log(*OR*)≈*d*×1.8138. Therefore, latent-scale differences of approximately 0.36, 0.91, and 1.45 correspond to conventional small (*d* = 0.2), medium (*d* = 0.5), and large (*d* = 0.8) effect sizes, respectively (Cohen, 1988). This same conversion applies to trend differences with covariates. Because our covariates were standardized, a trend difference represents the change in the latent-scale slope per one-standard-deviation change in the covariate. Dividing this trend difference by 1.81 converts the interaction into a standardized, *d*-like metric.

The performance of regression models was assessed by comparing them against the mean-only model and using standard Pearson *R*^2^ to compare against the boosting model, and the Bayesian version of *R*^2^ for ordinal data (Gelman et al., 2019). All models were multivariate, having all six evaluation criteria as outputs. Performance of models was evaluated by fitting the models with the training data and testing prediction accuracies with the testing dataset. Finally, the models were fit using all available data. The results are reported for these models, unless stated otherwise.

## 3 Results

### 3.1 Gradient boosted trees

After fine-tuning the hyperparameters with the training set, our best model reached correlation (*R*-value) of 0.68 (*R*^2^ = 0.46) for the training data and 0.59 (*R*^2^ = 0.35) for the test data. Corresponding *R*-values for individual outputs were: 0.63 (Suitability), 0.45 (Decision-making), 0.53 (Patriotism), 0.58 (Trustworthiness), 0.57 (Cooperation) and 0.60 (Righteousness). For individual politicians, R-values varied between 0.39 (SE) and 0.70 (JH). All R^2^ values are listed in the online Supplementary material 2 (Table S1).

**Figure 1:**
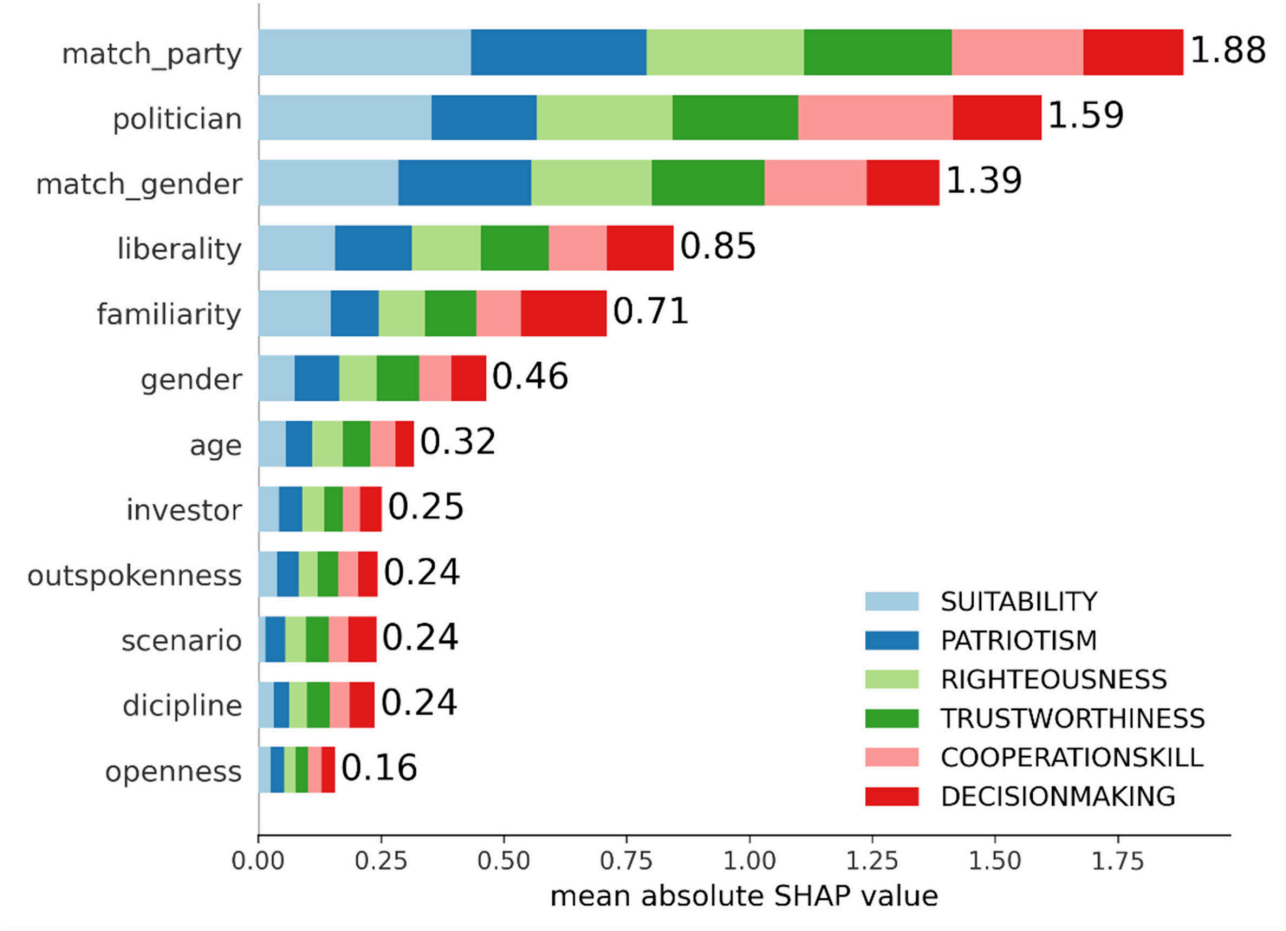
Relative importance of features in the boosting model. Feature importances are computed as SHAP values (total 19,962 for each bar) by taking absolute values and mean. Sub-bars and related colors depict the importance of individual output dimensions.

After the model was verified, we analyzed predictions for the test data. For this purpose, we computed SHAP values. One-hot encoded predictors of factors were combined by summing their SHAP values. Mean absolute SHAP values are depicted in Figure 1. The larger the absolute value, the higher the importance the variable has for prediction. The three most important variables were “match_party”, “politician”, and “match_gender”. The scenario, however, was the third least important variable. In other words, the scenario had only a minor impact on the responses with an average impact of around 0.04 (i.e., 4% of a single step in the Likert scale). This can be compared against “match_party” condition with an average impact around 0.31 (31%).

We also computed SHAP values for pair-wise interactions. The mean absolute interaction SHAP values are depicted in the online Supplementary material 2 (Figure S1). Top-9 interactions with values over 0.10 were as follows: politician:liberality (0.58), gender:match_gender (0.36), match_gender:familiarity (0.18), politician:investor (0.15), match_party:familiarity (0.13), gender:liberality (0.13), match_gender:liberality (0.13), politician:match_gender (0.12) and politician:familiarity (0.11). Again, the importance of the scenario was small, with the highest interaction being politician-scenario (0.03).

**Figure 2:**
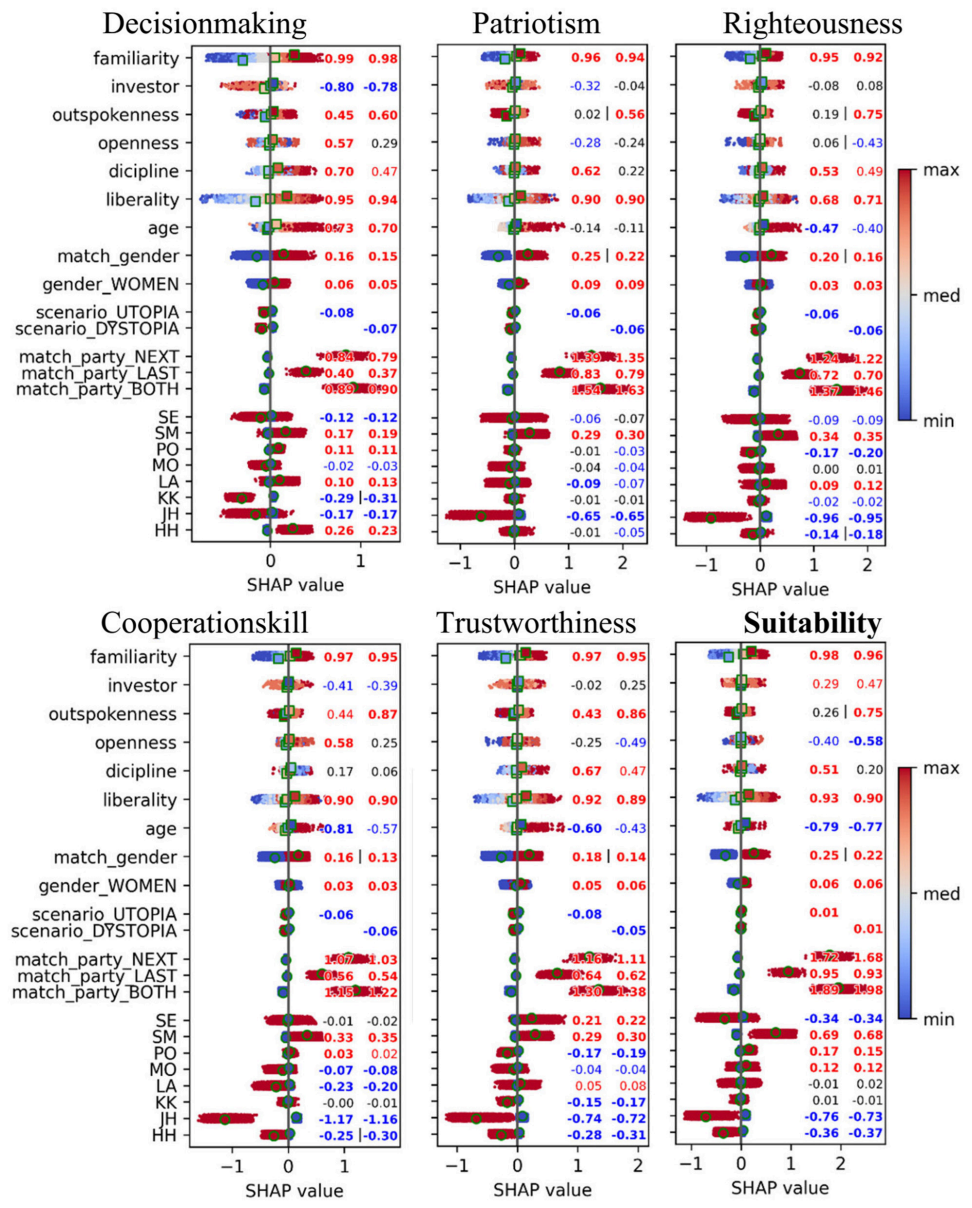
Impact of variables to the predictions of the boosting model. Each point here (total 19,962 for 1,109 subjects) represents SHAP value for one sample, where value 1 corresponds to single step in Likert-scale. The spheres correspond to category-specific mean values. Squares correspond to means of binned covariates (low, mean and high bins). Colored values depict *p* < 0.05, bolded values *p* < 0.001 and symbol “|” *p* < 0.05 for the comparison between the two, all computed using permutation tests (10,000 iterations). All *p*-values are adjusted with the FDR method. Note that reference levels for categorial variables are not shown.

Next, we inspected the SHAP values without pooling or taking absolute values. This allows a detailed view of how the individual variables contribute to the predictions, both negative and positive contributions. The data with a total 11096 × 2 × 9 × 1 = 19772 values are depicted in Figure 2 with all 22 variables. Note that, because of the one-hot encoding scheme, the following variables were set as the reference level: “AH” (for politician), “match_party = None” and “scenario=Baseline” and are not present in the results. As a guide for the eye, the figure also depicts the mean values for categories and binned numerical variables. For this purpose, numeric variables were binned into three bins (low, medium, high), each with 33% of the data.

We conducted a series of statistical tests for each variable to assess the robustness of its SHAP values. We only used the test data. The resulting test values, i.e., medians for factors and correlations for covariates, are included in Figure 2. Colored values depict *p* < 0.05 and bolded values *p* < 0.001. Symbol “|” indicates statistically significant difference at *p* < 0.05 between the two scenarios. Values were computed separately for Utopia and Dystopia. High/low correlation values indicate a positive/negative relationship between numeric trait scores and predicted output. If close to zero, there is no correspondence between the two. We found that SHAP values were highly robust in the sense that the majority of medians and covariates were statistically non-zero. We notice that the effect of the scenario was negative for all but Suitability. No difference between scenario types was found.

**Figure 3:**
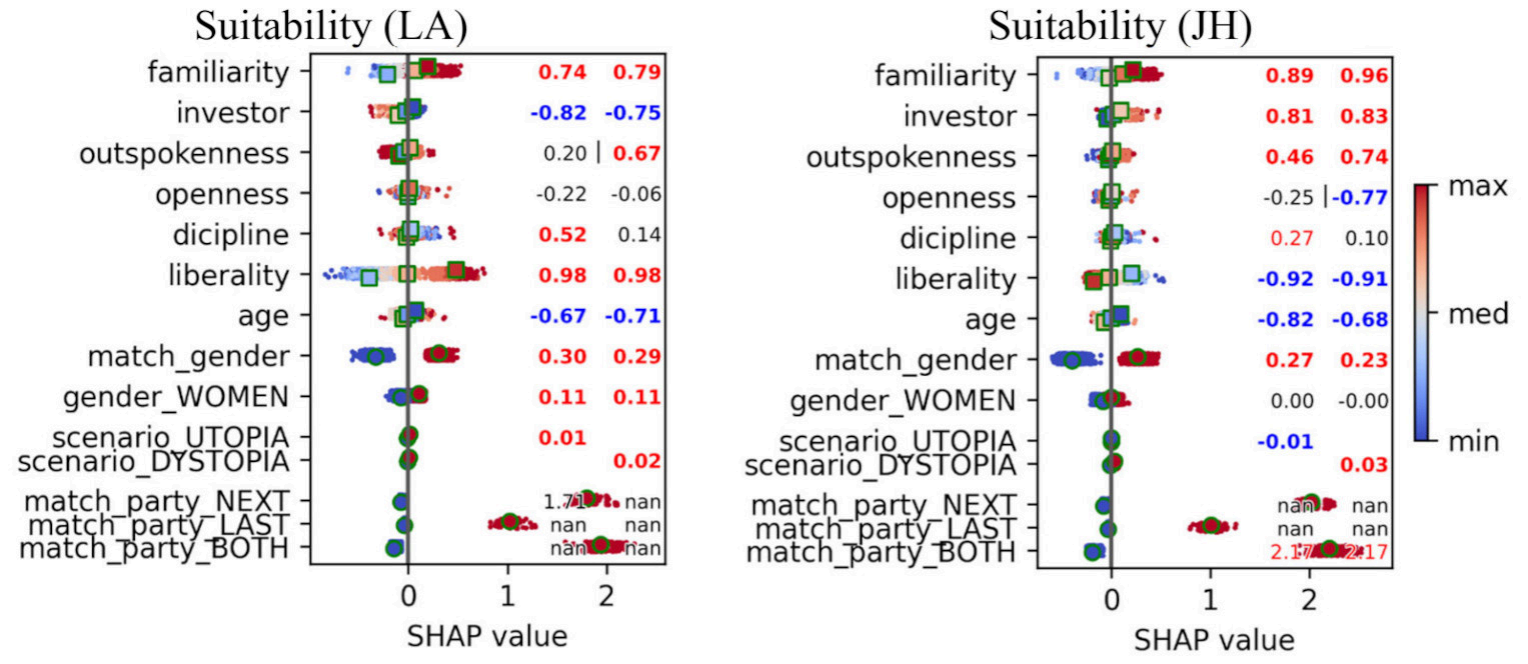
Impact of features to the predictions of the boosting model for JH and LA and response Suitability. Only samples for politicians LA **(left)** and JH **(right)** were analyzed. Each point here (total 19,962 for 1,109 subjects) represents SHAP value for one sample, where value 1 corresponds to single step in Likert-scale. Colored values depict *p* < 0.05, bolded values *p* < 0.001 and symbol “|” *p* < 0.05 for the comparison between the two, all computed using permutation tests (10,000 iterations). All *p*-values are adjusted with FDR. If less than five samples, no statistics were computed, and here shown as “nan”.

Next, we investigated two individual politicians. Figure 3 depicts results for the response Suitability for politicians JH and LA, which demonstrated two extreme cases. We see that “investor” and “liberality” had opposite impacts (different signs). Also, women favored LA (zero effect for JH) and predicted ratings in the utopia scenario were negative for JH. The impact of the matching party was higher for JH, indicating that voters of his party valued JH particularly highly.

**Figure 4:**
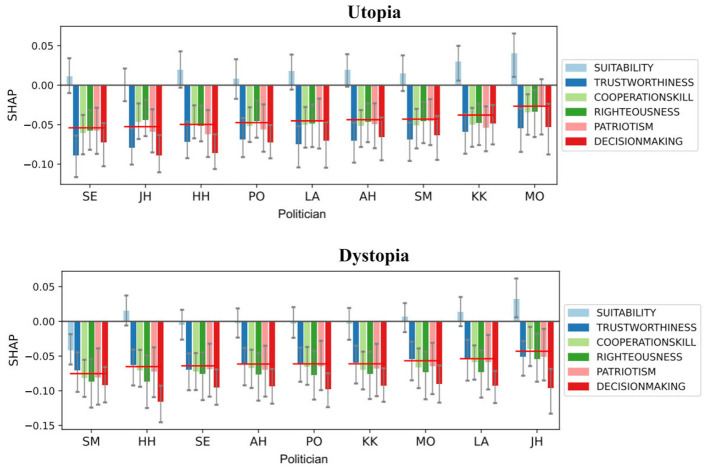
The politician-wise impact of utopia and dystopia scenarios. Each bar shows the mean SHAP value of the scenario column (utopia or dystopia) with 95th percentile limits. Politicians are sorted based on their grand average over all six responses. Here we used the boosting model with all data (*n* = 577 for utopia and *n* = 532 for dystopia).

Finally, in Figure 4, we depict the predicted SHAP values for utopia and dystopia for each politician separately. Here we consider all samples with their mean and 95th percentile limits. Results are sorted according to mean values over all six responses. Two findings are evident. Firstly, the mean of suitability is typically positive, while other responses are negative. Secondly, JH shifts from 8th in utopia to 1st in dystopia, which is the biggest change in rank (the second largest being 3 by LA).

### 3.2 Bayesian linear regression

The fitted Bayesian linear model for the testing data obtained *R*^2^ = 0.23, which was worse than the boosting model (0.35). This was expected due to linearity constraints and also because of the ordinal scale of outputs. On the other hand, the corresponding Bayesian *R*^2^ value of the model was 0.86 (95% HDI between 0.79 and 0.89), indicating a good fit.

In this analysis, we considered politicians as random effects variables and only considered the overall effects generalizing over all politicians. Individual random effects levels for each politician are depicted in the online Supplementary material (Figure S2), closely corresponding to the ranking obtained via gradient boosting. The fitting parameters of the model are listed in Supplementary material 3.

Next, we computed a series of contrasts to make inferences about the overall effect of variables and scenarios. Considering the large effect of the “match_party” already demonstrated for the boosting model (Figure 1), we separated predictions for the “match_party=None” and “match_party=Both or Next”. We considered the first group as *non-devoted voters* and the second as *devoted voters* of a specific political party. Without this separation, our results would provide the mean effect, which would lead to incorrect conclusions about the data. For the factors, we report estimated means and for the covariate's linear trends. These are computed separately for each of the six outputs and summarized as means over the outputs. All values are shown in the latent scale, i.e., the scale before transforming into numerical response scales via the cumulative model.

First, we computed marginal means at the baseline, i.e., parts A-C of the survey without scenarios. The results are shown in Table 3, separated between devoted and non-devoted voters. Here, the non-devoted males evaluating female politicians—“male (not_match)”—was the reference level, marking zero 0. Devotion had a major effect on evaluations with a large increase of means (over 1.45, with a large effect size) and trends, with 11 out of 13 being positive at 95% HDI, with only “investor” having a notable negative trend. For the non-devoted voters, covariate “liberality” and response “Decisionmaking” stand out by having a consistent positive effect; however, only few trends were above 0.36 (weak effect limit). Covariate “familiarity” had a strong positive trend with ratings for all responses and both devotion levels (over 1.45).

**Table 3:** Estimated marginal means and trends at the baseline for the linear model.

	**Non-devoted voters**							**Devoted voters**						
	**P**	**D**	**C**	**S**	**T**	**R**	**Mean**	**P**	**D**	**C**	**S**	**T**	**R**	**Mean**
[mean]	0.66	**1.42**	−0.61	0.22	−0.67	−0.72	0.05	**6.46**	**4.27**	**3.36**	**6.34**	**4.32**	**4.51**	**4.88**
**Condition**														
Male	0.42	1.30	−0.56	0.08	−0.78	−0.65	−0.03	**7.04**	**4.23**	**3.58**	**6.89**	**4.84**	**5.04**	**5.28**
Female	0.90	**1.55**	−0.66	0.37	−0.57	−0.79	0.14	**5.89**	**4.30**	**3.14**	**5.79**	**3.81**	**3.97**	**4.49**
Male (not match)	0^*^	0^*^	0^*^	0^*^	0^*^	0^*^	0^*^	**6.63**	**2.93**	**4.14**	**6.81**	**5.62**	**5.69**	**5.30**
Female (not match)	−0.50	1.65	−2.51	−1.36	−2.68	−2.97	−1.39	**4.49**	**4.41**	1.29	4.06	1.68	1.80	**2.96**
Male (match)	0.83	2.61	−1.13	0.15	−1.57	−1.30	−0.06	**7.46**	**5.54**	3.02	**6.97**	**4.06**	**4.39**	**5.25**
Female (match)	**2.29**	**1.44**	**1.19**	**2.10**	**1.56**	**1.39**	**1.66**	**7.28**	**4.20**	**5.00**	**7.51**	**5.92**	**6.15**	**6.01**
**Covariate**														
Age	0.15	0.17	0.00	−0.10	0.07	0.08	0.06	0.15	0.17	0.00	−0.10	0.07	0.08	0.06
Investor	−0.07	−0.11	−0.09	0.09	−0.02	−0.08	−0.05	−0.47	−0.27	−0.28	−0.33	−0.35	–**0.61**	−0.39
Liberality	**0.29**	**0.41**	0.22	**0.34**	**0.30**	0.14	**0.28**	0.08	**0.48**	0.00	0.06	−0.08	0.01	0.09
Dicipline	0.04	0.12	−0.05	−0.04	0.06	0.09	0.04	0.36	0.26	0.36	0.25	0.36	0.19	0.30
Openness	−0.06	0.00	0.00	−0.11	−0.07	−0.04	−0.04	0.31	0.25	0.28	0.36	0.39	0.45	0.34
Outspokenness	0.01	0.14	0.10	0.04	0.14	0.05	0.08	0.39	0.34	0.42	0.22	**0.53**	0.48	0.40
Familiarity	**0.23**	**0.62**	**0.46**	0.17	**0.41**	**0.36**	**0.37**	**2.94**	**2.19**	**2.35**	**3.05**	**3.01**	**2.90**	**2.74**

Values above the dotted line are means for factors and the those below are linear trends for covariates. Males evaluating female politicians “male (not_match)” was used as the reference level and fixed to zero. Colored values indicate statistical significance at level HDI >95% with color depicting positive (red) and negative (blue) sign and bolded values indicate HDI > 99.9% (both two-tailed). P, Patriotism; D, Decisionmaking; C, Cooperationskill; S, Suitability; T, Trustworthiness; R, Righteousness. ^*^Reference level.

Next, we studied the effect of the scenarios. For this, we computed contrasts for dystopia (D) and utopia (U) vs. the baseline (B), i.e., estimates “U – B” and “D – B” and their difference, the DID value. The results are listed in Table 4. Values close to zero indicate indifference to the scenario, while distinctly positive/negative values indicate a tendency for increased/decreased ratings compared to the baseline. Firstly, we noticed that for the non-devoted voters, both scenarios notably decreased ratings for all but Suitability, for which no effect was found. Most differences fell between low and medium effect sizes (0.36 and 0.91). The decrease was generally more substantial for dystopia. For the trends, notable differences between scenarios were found for the covariate “investor” for Patriotism, Cooperation skills, and Righteousness. However, all trends had a negligible effect size (< 0.36).

**Table 4:** Estimated means and linear trends of scenarios for the linear model (scenario contrast).

**Condition**	**Covariate**	**Patriotism**		**Decisionmak**.		**Cooperation**.		**Suitability**		**Trustworth**.		**Righteous**.		**Mean**	
		**Dys**.	**Uto**.	**Dys**.	**Uto**.	**Dys**.	**Uto**.	**Dys**.	**Uto**.	**Dys**.	**Uto**.	**Dys**.	**Uto**.	**Dys**.	**Uto**.
**Non-devoted voters**															
[mean]		**−0.52** ^*^	**−0.36**	**−0.71** ^*^	**−0.57**	**−0.44**	**−0.36**	−0.05^*^	0.09	**−0.50**	**−0.40**	**−0.59** ^***^	**−0.32**	**−0.47** ^***^	**−0.32**
Male		**−0.55**	**−0.40**	**−0.76**	**−0.61**	**−0.54**	**−0.44**	−0.07^*^	0.14	**−0.57** ^*^	**−0.36**	**−0.73** ^***^	**−0.37**	**−0.54** ^***^	**−0.34**
Female		**−0.50** ^*^	**−0.33**	**−0.67**	**−0.54**	**−0.35**	**−0.28**	−0.03	0.03	**−0.44**	**−0.43**	**−0.46** ^*^	**−0.27**	**−0.41** ^**^	**−0.30**
Male	Investor	0.12^*^	−0.06	−0.13	−0.02	0.07	−0.01	0.01	0.00	0.00	0.00	0.15^*^	0.01	0.04	−0.01
Female		0.20^**^	−0.10	−0.05	−0.03	0.24^**^	−0.05	0.08	−0.10	0.13^**^	−0.15	0.24^***^	−0.13	**0.14** ^***^	**−0.09**
Male	Liberality	−0.01	−0.08	−0.02	0.01	−0.05	0.05	−0.07	−0.05	0.07	−0.02	0.02	−0.02	−0.01	−0.02
Female		−0.01	−0.16	0.04	−0.10	−0.02	−0.09	−0.19	−0.12	−0.09	−0.05	−0.05	−0.07	−0.06	**−0.10**
Male	Dicipline	0.05	0.03	−0.02	−0.15	0.02	0.02	0.04	0.05	−0.11^*^	0.06	−0.02	−0.10	−0.01	−0.02
Female		−0.07	0.00	0.04	−0.03	0.02	0.00	−0.04	−0.04	0.07	0.01	−0.05	−0.01	0.00	−0.01
Male	Openness	0.13	0.03	0.14	0.01	0.16^*^	−0.03	0.08	0.07	0.02	0.01	0.06	0.08	0.10	0.03
Female		0.01	0.00	−0.07	−0.06	−0.03	0.07	−0.02	0.07	0.06	0.12	0.09	0.02	0.01	0.04
Male	Outspokenness	0.02	−0.06	0.16	0.04	−0.08	0.00	0.01	0.02	−0.01	−0.04	−0.04	−0.12	0.01	−0.03
Female		0.00	−0.03	0.02	−0.03	0.03	−0.07	0.00	0.00	0.02	−0.07	0.02	−0.07	0.01	−0.05
Male	Familiarity	0.08	0.06	−0.17	−0.12	−0.13	−0.16	−0.05	−0.03	−0.18	−0.08	−0.15	−0.07	**−0.10**	−0.07
Female		−0.05	−0.01	−0.17	−0.06	−0.14	−0.09	0.01	0.00	−0.06	−0.14	−0.03	−0.09	−0.07	−0.06
**Devoted voters**															
[mean]		−0.27	0.09	−0.24	0.05	0.28	0.48	0.26^**^	**0.81**	−0.13^*^	0.39	0.12	0.37	0.00^***^	**0.37**
Male		−0.56	0.01	−0.28	−0.02	0.29	0.44	0.31	0.75	−0.37^*^	0.46	0.28	0.48	−0.06^*^	0.35
Female		0.01	0.18	−0.19	0.12	0.27	0.51	0.20^**^	**0.88**	0.11	0.33	−0.03	0.26	0.06^**^	**0.38**
Male	Investor	0.36	−0.09	−0.04	0.21	−0.18	−0.08	−0.01	0.10	0.10	−0.05	0.20	0.06	0.07	0.03
Female		0.11	−0.44	−0.40	−0.27	0.14	−0.20	−0.34	−0.48	−0.26	−0.29	−0.15	−0.16	−0.15	−**0.31**
Male	Liberality	−0.11	−0.04	−0.36	−0.14	0.10	0.04	0.08	−0.23	0.27	0.20	0.17	0.10	0.03	−0.01
Female		−0.19	0.34	−0.14	0.42	0.10	0.42	0.05	0.28	0.34	0.41	−0.24	0.24	−0.01^**^	**0.35**
Male	Dicipline	0.09	0.36	−0.23	−0.03	−0.13	−0.17	0.15	0.28	−0.31	0.18	−0.18	0.18	−0.10	0.13
Female		−0.13	−0.05	0.08	0.13	0.06	−0.26	0.16	−0.03	0.11	0.09	0.02	0.10	0.05	−0.01
Male	Openness	0.10	−0.39	0.10	−0.32	0.21	−0.23	0.16	−0.30	−0.34	−0.15	0.02	−0.43	0.04^*^	−0.30
Female		0.30	0.15	0.37	−0.06	0.17	0.13	0.22	0.26	−0.14	0.16	0.38	0.23	0.22	0.14
Male	Outspokenness	−0.46^*^	0.30	0.02	0.29	−0.21^*^	0.44	−0.59^**^	0.43	−0.26	−0.05	−0.18	0.06	−0.28^**^	0.25
Female		0.10	0.04	0.07	0.11	−0.17	−0.20	0.47	0.17	0.37	−0.02	0.20	0.12	0.17	0.04
Male	Familiarity	0.03	−0.44	−0.38	−0.05	−0.60	−0.34	−0.03	−0.63	−0.49	−0.58	−0.41	−0.08	−0.31	−0.35
Female		−0.20	−0.45	−0.14	−0.17	−0.19	−0.13	−0.71	−0.63	−0.25	−0.25	−0.25	−0.50	−0.29	−**0.36**

Each value is an estimated change against the baseline for Utopia (U) and Dystopia (D) with respect to devotion level, gender and covariates. For factors, positive values indicate an increase in rating in the corresponding scenario (decrease for negative values). For covariates, the values represent linear trends. Colored values indicate statistical significance at level HDI >95% with color depicting positive (red) and negative (blue) sign and bolded values indicate HDI > 99.9% (both two-tailed). Stars indicate DID comparisons between the two scenarios with^*^ = 95%, ^**^ = 99% and ^***^ = 99.9% HDI (two-tailed).

For the devoted voters, the scenario's impact was much weaker, with very few values surpassing the 95% HDI threshold. Utopia increased the valuations for all but Patriotism and Decision-making. Effect sizes we typically between low and medium, also *between* scenarios. Interestingly, covariate “familiarity” had a notable negative trend under all conditions, i.e., both genders, devotion levels, and scenarios. The more familiar the politician, the lower the valuation was in both scenarios. All trends had effect sizes between low and medium, hence larger compared to non-devoted voters.

To give a rough estimate of whether the scenario influenced covariates, we may count how many times the trend surpassed HDI 95% for non-zero. Counts for non-devoted were (male/female): 3/4 for the investor, 1/3 for liberality, 1/0 for discipline, 2/1 for openness, 1/0 for outspokenness, and 4/3 for familiarity. Counts for devoted voters: 0/2 for investors, 0/2 for liberality, 0/0 for discipline, 0/0 for openness, 1/2 for outspokenness, and 3/4 for familiarity.

Finally, we directly compared the effects of gender and devotion level. Results are shown in Table 5. Here, for simplicity, we only consider the mean over the six responses. Positive (red) values indicate a larger scenario-induced change for female and devoted voters. We notice that ratings were, on average, smaller for males for both devoted and non-devoted voters. However, only the difference for non-devoted voters in Dystopia surpassed 95% HDI with a negligible effect size. For devotion level, effect sizes were between weak and medium. For trends, there were significant average differences for all but “discipline” and “familiarity” covariates at 95% HDI or more. The largest differences (at 99.9% HDI) were found for “openness” and “outspokenness” where devote female voters had stronger trends for Utopia (for “openness”) and Dystopia (for “outspokenness”). Weak effect sizes were present for liberality, openness and outspokenness, both for individual scenarios and their differences.

**Table 5:** Devotion and gender-induced valuation change under the scenario for the linear model.

		**Mean**		**Mean**		**Mean**		**Mean**	
		**Dys**.	**Uto**.	**Dys**.	**Uto**.	**Dys**.	**Uto**.	**Dys**.	**Uto**.
**Covariate**	[mean]	0.13	0.04	0.12	0.03	**0.47**	**0.68**	**0.48**	**0.69**
	Investor	0.10^**^	−0.08	−0.22	−0.33	−0.29	−0.21	0.03	0.04
	Liberality	−0.05	−0.08	−0.04^*^	0.36	0.04^**^	**0.45**	0.04	0.00
	Dicipline	0.01	0.01	0.15	−0.14	0.05	0.01	−0.09	0.15
	Openness	−0.09	0.01	0.18	**0.45**	0.21	0.11	−0.06	**−0.34**
	Outspokenness	0.00	−0.02	**0.45** ^***^	−0.21	0.16	0.08	−0.29^***^	0.27
	familiarity	0.03	0.00	0.02	0.00	−0.22	−0.29	−0.21	−0.29
	Condition:	Non-devoted		Devoted		Female		Male	
	Contrast:	Female—Male		Female—Male		Devoted—non-devoted		Devoted—non-devoted	

Each value is an estimated difference of difference against the baseline for Utopia (U) and Dystopia (D) computed as the mean over all responses. Columns 1 and 2 show gender contrast for non-devoted (1st) and devoted (2nd) voters. Columns 3 and 4 show devotion contrast for females (3rd) and males (4th). Colored values indicate statistical significance at level HDI >95% with color depicting positive (red) and negative (blue) sign and bolded values indicate HDI > 99.9% (both two-tailed). Positive values indicate larger scenario-induced change for females (columns 1 and 2) and devoted voters (columns 3 and 4). Stars indicate comparisons between the two scenarios with ^*^ = 95%, ^**^ = 99% and ^***^ = 99.9% HDI (two-tailed).

## 4 Discussion

In this study, we investigated how electorates evaluate political candidates for Prime Minister when exposed to hypothetical near-future utopian and dystopian scenarios. Our findings are based on an online survey in which 1,109 Finnish electorates evaluated nine Finnish politicians on six attributes, including patriotism, decision-making, cooperation skills, trustworthiness, righteousness, and suitability as a prime minister. By applying both machine learning and Bayesian statistical methods to this data, we analyzed the interplay between voter characteristics, politician attributes, and contextual framing (scenario) on these valuations. The results provide a multifaceted view of the factors that shape political judgment in a multi-party system.

The central aim of this study was to assess how contextual utopian and dystopian scenarios influence voters' evaluations. Overall, the impact of the scenario was found to be minor compared to other predictors in our models. As measured with SHAP values, the scenario was the 10th most important predictor out of all 12 feature categories with almost an eight-fold difference to the most important feature of matching party (see Figures 1, 2). A similar pattern appeared in the Bayesian model, where scenario-related differences in mean intercepts were roughly 12-fold smaller (Tables 3, 4). Effect sizes between scenarios and the baseline were between weak and moderate, while effect sizes between the two scenarios were typically negligible. This effect was weaker than we expected. We assumed that narratives would activate strong emotional, future-oriented mental models influencing candidate evaluations. Typically, when an individual reads the scenario text, s/he uses mainly a conscious strategy (Stanovich and West, 2000; Thomadsen et al., 2018). Then it may be that our texts alone lacked impact, as if the same scenarios were presented by narrators with images, videos, or audio (Schmälzle et al., 2014; Tikka et al., 2018). Moreover, narratives grounded in expert-elicited scenarios could have enhanced their persuasive and realistic impact (*cf*. Edlmann et al., 2016).

Despite the weak overall effect, the scenarios produced distinct patterns of change. For non-devoted voters, both utopia and dystopia scenarios led to a general decrease in ratings across other ratings except suitability. This decrease was, on average, more pronounced with four out of six attributes in the dystopia scenario (Table 4). For devoted voters, ratings generally increased for the utopia (four out of six responses and on average) while no effect was found for dystopia. It might be the case that non-devoted voters felt choice fatigue (Augenblick and Nicholson, 2016), reflected in the second round of evaluations as a decreasing rating, while devoted voters had higher rating stability and less susceptibility to fatigue and contextual framing. Finally, the effect of the scenario was similar between genders for the means. Notable difference was found only in non-devoted voters in dystopia scenario, where the decrease in ratings by men was stronger (−0.54 for men vs. −0.41 for women at latent scale; Table 4). The difference was largest (0.27) for the righteousness attribute.

When looking at individual politicians, we found the valuation of the right-wing politician JH increasing substantially in the dystopian scenario, moving his rank from eighth in utopia to first in dystopia (see Figure 4). This provides empirical support for the hypothesis that in times of perceived crisis or instability, a segment of the electorate may gravitate toward leaders perceived as ideologically decisive. Our analysis further showed that voter traits such as liberality and risk-taking propensity (investor) had opposite effects on the valuation of politicians at opposite ends of the political spectrum (JH and LA, see Figure 3), demonstrating the utility of ML-based modeling for uncovering such specific patterns.

Beyond their effects on overall candidate rankings, the scenarios also altered the underlying structure of evaluation by changing how voters weighed certain traits. At the baseline (no scenarios), liberality and familiarity had strong positive trends for non-devoted voters (see Table 3). For devoted voters, traits such as discipline, openness, outspokenness, and familiarity showed strong positive trends, while investing showed the opposite pattern. Familiarity increased ratings in both groups, though much more strongly among devoted voters (slope 2.74 vs. 0.37). When comparing scenario-based slopes to baseline, several significant changes (HDI>95% or higher) emerged depending on voter devotion, gender, and evaluation criteria (Table 4). For non-devoted voters, more liberal voters gave higher ratings, while the effect disappeared for devoted voters. For devoted voters, high openness, outspokenness and discipline with a low investor score increased ratings.

From these results, two conclusions can be drawn. Firstly, dystopia and utopia had few notable effects for all covariates except discipline. For example, higher investor scores for women decreased ratings in utopia regardless of devotion level. Also, a higher liberality score for women decreased ratings in utopia for non-devoted voters, while the effect was opposite for devoted voters (Table 4). A notable gender difference was found for devoted voters for openness in utopia and outspokenness in dystopia with opposite trends between men and women (Table 5). Secondly, dystopia and utopia reduced the effect of familiarity: Dependency on politician familiarity was pushed closer to zero (Table 4). While high familiarity still increased ratings (trend remained positive), its effect was notably diminished for scenarios. Interestingly, this reduction effect was stronger for devoted voters (Table 5).

Our regression models identified party devotion as the primary driver of candidate evaluation. A voter's past or intended future vote for a politician's party substantially boosted their ratings across all attributes (see Figures 1, 2, and Table 3). While this aligns with expectations, its strength relative to other factors is notable. This result is consistent with the “spillover” phenomenon, where voters committed to a party or politician tend to value their positions favorably, regardless of other considerations (Marks et al., 2019). This underscores the necessity of distinguishing between devoted and non-devoted voters when interpreting regression models and their predictions.

Beyond partisanship, the gender match between a voter and a candidate also emerged as a powerful predictor. Measured via SHAP, the effect of matching gender was almost as strong as the politician itself (see Figure 1). Female respondents tended to give higher ratings to female politicians (see Table 3), a finding that corroborates previous research on Finnish elections (Giger et al., 2014; Kauttonen and Suomala, 2019) and in the USA (Brians, 2005).

The dual-analytic approach, utilizing gradient boosting and Bayesian linear modeling, employed in this study was instrumental in revealing these layered findings. The two computational approaches complemented each other by having different underlying assumptions and limitations. The real-world interplay of factors in how the electorate evaluates politicians is inherently complex, and our methodology was chosen to explore this complexity rather than reduce it with strong *a priori* assumptions. The gradient boosting model excelled at identifying the most powerful predictors and non-linear relationships, while the Bayesian linear mixed-effects model provided the inferential depth needed to dissect the nuanced interaction effects and quantify their uncertainty. We argue that combining these complementary approaches allows the data to be interrogated more fully, and we advocate for the wider adoption of such integrated workflows in political science. The impact of the scenarios could be diversified in future studies by allowing participants to freely describe the thoughts and feelings evoked by the scenarios. These responses could then be analyzed using natural language processing (NLP) methods (Schmälzle et al., 2014).

Overall, our work makes two main contributions to existing research. First, we investigated the effect of realistic future scenarios on multi-dimensional valuations of well-known politicians. We pinpointed phenomena that affected the valuations related to both electorates and politicians. While some of the results were expected, such as strong gender and partisan dependency, others were novel, such as both positive and negative contextual future scenarios decreasing politician ratings and the effect of familiarity being diminished in scenarios. We also noticed other subtle effects, such as men being more sensitive to opinion change in scenarios and outspokenness having notably different covarying trends for scenarios depending on gender and party devotion. Secondly, we applied a dual-analytic approach to leverage both high interpretability and statistical inference of linear models with higher predictive power with the inclusion of nonlinear effects of machine learning (ML). We further promote introducing ML-based workflows of political research to improve understanding and predictions for citizens' voting behavior.

We acknowledge certain limitations in this work. Firstly, comparing the actual number of votes received by the politicians and their parties (Table 1) with the relative valuation of our nine politicians (see Figure 4 and the online Supplementary material (Figure S2) suggests that our sample does not represent the Finnish population accurately. However, as the purpose of this work was not to replicate the actual popularity of politicians *per se*, we don't consider this limitation a severe one. Secondly, the number of politicians was only nine, which was set by the number of major parties in Finland and the time constraints of our survey. Our rationale here was that the heads of political parties were well known by the population, which allowed more throughout evaluation of the politicians. Thirdly, the background information of responders involved only 10 variables (resulting in 8 model features). While collecting more detailed information, e.g., the responders' personality and voting history, might improve the predictive power of models, it would have had a negative impact on data collection (longer surveys) and willingness to participate (sensitivity of data). Fourthly, asking participants to rate each politician twice with six different aspects can result in survey fatigue, particularly toward the end. Our methods to avoid the negative effect of this were to keep the survey concise with only one scenario per subject and to randomize candidate order. Finally, in an ideal situation, we would monitor actual longitudinal voting behavior and collect a more comprehensive set of parameters. However, this was not feasible because of secret ballot voting in Finland and the practical difficulties of collecting such sensitive data.

## 5 Conclusion

We investigated how two hypothetical utopia and dystopia scenarios affected the electorate's valuation of nine leading Finnish political candidates in Finland. We found that the strongest predictors for the electorate's valuation were the party, gender, and familiarity of the candidate, as well as liberal or conservative attitudes of the voter. The effect of scenarios was found to be relatively weak with an overall tendency to reduce the valuations of politicians, and was also associated with a complex interplay with other variables. While high familiarity with a politician increased ratings at baseline, scenarios diminished this dependency. Also, devoted voters were less likely to change their opinion. When looking at individual politicians, we found an increased valuation toward a right-wing politician in a dystopian scenario. The scenario had a mostly similar effect regardless of gender. However, we found that liberality was associated with higher sensitivity to opinion change for women. We also demonstrated the importance of using complementary computational models in revealing different properties of the complex behavioral data related to politician evaluations.

Overall, our key findings were that *stable voter traits dominate candidate evaluation*. Party loyalty and gender match were the strongest predictors, showing how resilient core political judgments are to short-term framing. *Narratives, in turn, had complex and multifaceted effects*. The scenarios showed a subtle decrease in opinions. They interacted with partisanship by decreasing ratings among non-devoted voters, while also increasing the valuation of a right-wing candidate in a perceived crisis (dystopia). Finally, *leveraging complementary models is essential*. In our case, combining predictive machine learning with inferential statistics allowed us to identify both the primary drivers of voter judgements and the more nuanced interactions shaping voter cognition.

Our study brings new insight into context-dependent behavior of voting in a multi-party system and has timely implications for understanding political behavior in an era of uncertainty and polarization. Future research should expand on these findings by employing more immersive experimental designs beyond text, such as virtual reality or video narratives, to test the boundaries of contextual influence. Cross-cultural studies are also needed to determine if these patterns of judgment hold in different political systems, particularly two-party vs. multi-party democracies, and across different multiparty democracies with varying political cultures. Ultimately, this work highlights the need to move beyond simple main effects to explore the complex interactions that shape how citizens perceive their political leaders, a critical task for navigating the modern political landscape.

## Data Availability

The original contributions presented in the study are included in the article/Supplementary material, further inquiries can be directed to the corresponding author.
