# Supplementary material for: Behavioral science of voting in hypothetical utopia and dystopia scenarios: a predictive modeling approach
Source: Front Psychol. 2026 Jan 12;16:1713314. doi: 10.3389/fpsyg.2025.1713314 (PMC12833430; doi:10.3389/fpsyg.2025.1713314)
Supplement: Supplementary file 1 [file Data_Sheet_1.pdf]

Kyselyssä arvioidaan Suomen istuvan eduskunnan puoluejohtajia. Kysely on suomenkielinen ja sen täyttäminen kestää noin 15 minuuttia.

Kaikkien vastanneiden kesken arvotaan 20 kappaletta SOK-ryhmän lahjakortteja. Lahjakortin arvo on 25 € ja arvontaan osallistuminen edellyttää koko kyselyn täyttämistä ja sähköpostin antamista.

Kyselyaineisto käsitellään ja säilytetään siten, ettei yksittäisen vastaajan tiedot paljastu.

Kiitos osallistumisesta tutkimukseen!

Kysely alkaa!

There are 110 questions in this survey.

Taustatiedot

Aluksi kysymme muutamia taustatietoja.

Mikä on ikäryhmäsi? *

Valitse sopivin vaihtoehto:

	18-21	22-25	26-29	30-39	40-49	50-59	60+
**Valitse sopivin vaihtoehto**	◯	◯	◯	◯	◯	◯	◯

Mikä on sukupuolesi? *

Valitse sopivin vaihtoehto:

	Mies	Nainen	Muu / en halua vastata
**Valitse sopivin vaihtoehto**	◯	◯	◯

Seuraavassa esitetään väitteitä. Arvioi, kuinka hyvin väite sopii sinuun. *

Valitse sopivin vaihtoehto:

	1 Vahvasti eri mieltä	2 Kohtalaisesti eri mieltä	3 Hieman eri mieltä	4 Neutraali	5 Hieman samaa mieltä	6 Kohtalaisesti samaa mieltä	7 Vahvasti samaa mieltä
**Olen kurinalainen**	◯	◯	◯	◯	◯	◯	◯
**Olen huoleton**	◯	◯	◯	◯	◯	◯	◯
**Olen avoin uusille kokemuksille**	◯	◯	◯	◯	◯	◯	◯
**Olen tavanomainen**	◯	◯	◯	◯	◯	◯	◯
**Olen yhteiskunnallisesti konservatiivi**	◯	◯	◯	◯	◯	◯	◯
**Olen yhteiskunnallisesti liberaali**	◯	◯	◯	◯	◯	◯	◯
**Puolustan uskomaani epäsuosittua asiaa sosiaalisessa tilanteessa**	◯	◯	◯	◯	◯	◯	◯
**Investoin 5% vuosituloistani hyvin spekulatiivisiin osakkeisiin**	◯	◯	◯	◯	◯	◯	◯

Jos haluat osallistua arvontaan, anna sähköpostiosoite. Otamme yhteyttä mahdollisen voiton yhteydessä. Tietoa ei käytetä muuhun tarkoitukseen.

Vastauksesi:

Minulle on kerrottu riittävästi tutkimuksen perusideasta ja ymmärrän, että siihen osallistuminen on vapaaehtoista. *

Valitse **kaikki** jotka soveltuvat:

☐ Kyllä

{if (RandomScene.value>=1,RandomScene.value,rand(1,2))}

Part1page1

Suomi vuonna 2020

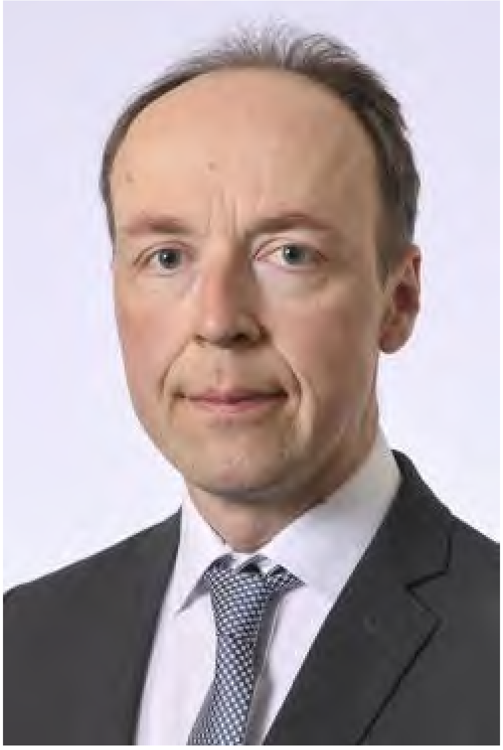

Jussi Halla-aho, PS

Kuinka tuttu tämä poliitikko on sinulle? Arvioi työtä poliitikkona älä yksityishenkilönä. *

Valitse sopivin vaihtoehto:

	1 Ei lainkaan	2	3	4 Jonkin verran	5	6	7 Erittäin tuttu
**Valitse sopivin vaihtoehto**	○	○	○	○	○	○	○

arvioi työtä poliitikkona, ei yksityiselämää

Vastaa väittämään: Hän on sopiva Suomen pääministeriksi. *

Valitse sopivin vaihtoehto:

	1 Vahvasti eri mieltä	2	3	4 Neutraali	5	6	7 Vahvasti samaa mieltä
**Valitse sopivin vaihtoehto**	◯	◯	◯	◯	◯	◯	◯

Arvioi ehdokasta vastaamalla seuraaviin väittämiin: *

Valitse sopivin vaihtoehto:

	1 Vahvasti eri mieltä	2	3	4 Neutraali	5	6	7 Vahvasti samaa mieltä
**Hän on luotettava**	◯	◯	◯	◯	◯	◯	◯
**Hän on yhteistyökykyinen**	◯	◯	◯	◯	◯	◯	◯
**Hän on oikeudenmukainen**	◯	◯	◯	◯	◯	◯	◯
**Hän toimii Suomen hyväksi**	◯	◯	◯	◯	◯	◯	◯
**Hän on päätöskykyinen**	◯	◯	◯	◯	◯	◯	◯

Kuvittele, että hän on uusi pääministeri. Mikä on valinnan herättämä tunne sinussa? *

Valitse sopivin vaihtoehto:

	1 Erittäin epämiellyttävä	2	3	4 Neutraali	5	6	7 Erittäin miellyttävä
**Valitse sopivin vaihtoehto**	◯	◯	◯	◯	◯	◯	◯

Miten suuri tämän tunteen voimakkuus on? *

Valitse sopivin vaihtoehto:

	1 Erittäin pieni	2	3	4 Kohtalaisen suuri	5	6	7 Erittäin suuri
**Valitse sopivin vaihtoehto**	◯	◯	◯	◯	◯	◯	◯

Part1page2

Suomi vuonna 2020

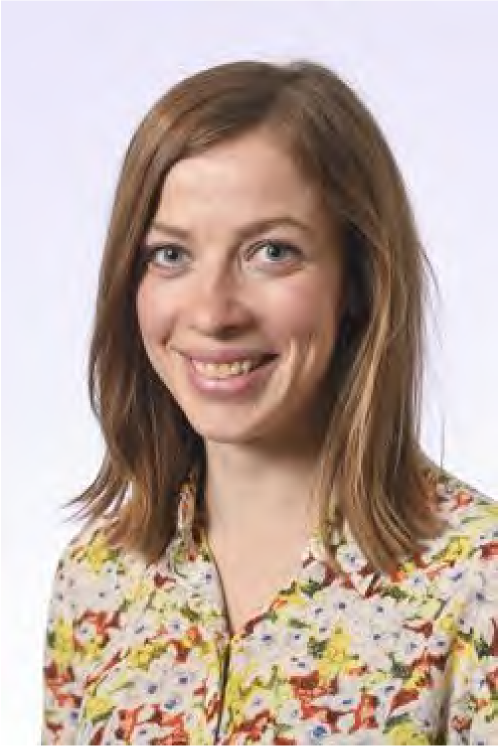

Li Andersson, Vas.

Kuinka tuttu tämä poliitikko on sinulle? Arvioi työtä poliitikkona älä yksityishenkilönä. *

Valitse sopivin vaihtoehto:

	1 Ei lainkaan	2	3	4 Jonkin verran	5	6	7 Erittäin tuttu
**Valitse sopivin vaihtoehto**	◯	◯	◯	◯	◯	◯	◯

arvioi työtä poliitikkona, ei yksityiselämää

Vastaa väittämään: Hän on sopiva Suomen pääministeriksi. *

Valitse sopivin vaihtoehto:

	1 Vahvasti eri mieltä	2	3	4 Neutraali	5	6	7 Vahvasti samaa mieltä
**Valitse sopivin vaihtoehto**	○	○	○	○	○	○	○

Arvioi ehdokasta vastaamalla seuraaviin väittämiin: *

Valitse sopivin vaihtoehto:

	1 Vahvasti eri mieltä	2	3	4 Neutraali	5	6	7 Vahvasti samaa mieltä
**Hän on luotettava**	○	○	○	○	○	○	○
**Hän on yhteistyökykyinen**	○	○	○	○	○	○	○
**Hän on oikeudenmukainen**	○	○	○	○	○	○	○
**Hän toimii Suomen hyväksi**	○	○	○	○	○	○	○
**Hän on päätöskykyinen**	○	○	○	○	○	○	○

Kuvittele, että hän on uusi pääministeri. Mikä on valinnan herättämä tunne sinussa? *

Valitse sopivin vaihtoehto:

	1 Erittäin epämiellyttävä	2	3	4 Neutraali	5	6	7 Erittäin miellyttävä
**Valitse sopivin vaihtoehto**	◯	◯	◯	◯	◯	◯	◯

Miten suuri tämän tunteen voimakkuus on? *

Valitse sopivin vaihtoehto:

	1 Erittäin pieni	2	3	4 Kohtalaisen suuri	5	6	7 Erittäin suuri
**Valitse sopivin vaihtoehto**	◯	◯	◯	◯	◯	◯	◯

Part1page3

Suomi vuonna 2020

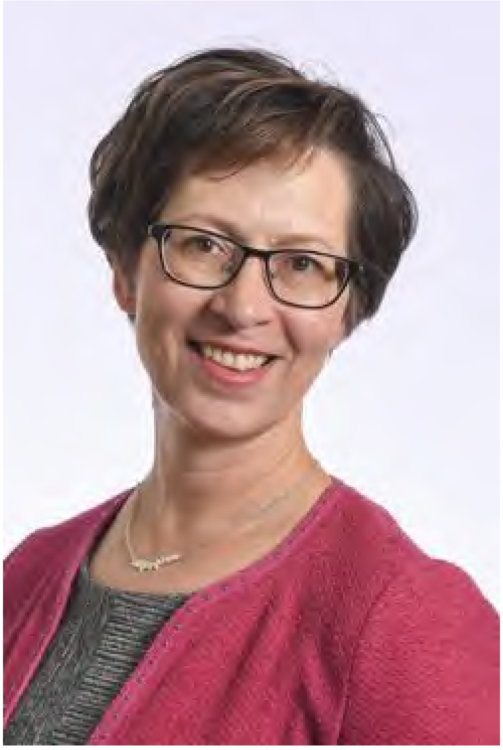

Sari Essayah, KD

Kuinka tuttu tämä poliitikko on sinulle? Arvioi työtä poliitikkona älä yksityishenkilönä. *

Valitse sopivin vaihtoehto:

	1 Ei lainkaan	2	3	4 Jonkin verran	5	6	7 Erittäin tuttu
**Valitse sopivin vaihtoehto**	◯	◯	◯	◯	◯	◯	◯

arvioi työtä poliitikkona, ei yksityiselämää

Vastaa väittämään: Hän on sopiva Suomen pääministeriksi. *

Valitse sopivin vaihtoehto:

	1 Vahvasti eri mieltä	2	3	4 Neutraali	5	6	7 Vahvasti samaa mieltä
**Valitse sopivin vaihtoehto**	◯	◯	◯	◯	◯	◯	◯

Arvioi ehdokasta vastaamalla seuraaviin väittämiin: *

Valitse sopivin vaihtoehto:

	1 Vahvasti eri mieltä	2	3	4 Neutraali	5	6	7 Vahvasti samaa mieltä
**Hän on luotettava**	◯	◯	◯	◯	◯	◯	◯
**Hän on yhteistyökykyinen**	◯	◯	◯	◯	◯	◯	◯
**Hän on oikeudenmukainen**	◯	◯	◯	◯	◯	◯	◯
**Hän toimii Suomen hyväksi**	◯	◯	◯	◯	◯	◯	◯
**Hän on päätöskykyinen**	◯	◯	◯	◯	◯	◯	◯

Kuvittele, että hän on uusi pääministeri. Mikä on valinnan herättämä tunne sinussa? *

Valitse sopivin vaihtoehto:

	1 Erittäin epämiellyttävä	2	3	4 Neutraali	5	6	7 Erittäin miellyttävä
**Valitse sopivin vaihtoehto**	◯	◯	◯	◯	◯	◯	◯

Miten suuri tämän tunteen voimakkuus on? *

Valitse sopivin vaihtoehto:

	1 Erittäin pieni	2	3	4 Kohtalaisen suuri	5	6	7 Erittäin suuri
**Valitse sopivin vaihtoehto**	◯	◯	◯	◯	◯	◯	◯

Part1page4

Suomi vuonna 2020

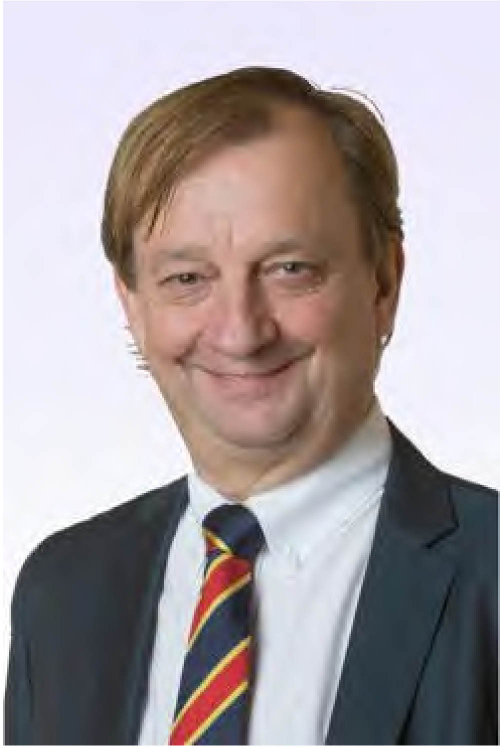

Harry Harkimo, Liike Nyt

Kuinka tuttu tämä poliitikko on sinulle? Arvioi työtä poliitikkona älä yksityishenkilönä. *

Valitse sopivin vaihtoehto:

	1 Ei lainkaan	2	3	4 Jonkin verran	5	6	7 Erittäin tuttu
**Valitse sopivin vaihtoehto**	○	○	○	○	○	○	○

arvioi työtä poliitikkona, ei yksityiselämää

Vastaa väittämään: Hän on sopiva Suomen pääministeriksi. *

Valitse sopivin vaihtoehto:

	1 Vahvasti eri mieltä	2	3	4 Neutraali	5	6	7 Vahvasti samaa mieltä
**Valitse sopivin vaihtoehto**	○	○	○	○	○	○	○

Arvioi ehdokasta vastaamalla seuraaviin väittämiin: *

Valitse sopivin vaihtoehto:

	1 Vahvasti eri mieltä	2	3	4 Neutraali	5	6	7 Vahvasti samaa mieltä
**Hän on luotettava**	○	○	○	○	○	○	○
**Hän on yhteistyökykyinen**	○	○	○	○	○	○	○
**Hän on oikeudenmukainen**	○	○	○	○	○	○	○
**Hän toimii Suomen hyväksi**	○	○	○	○	○	○	○
**Hän on päätöskykyinen**	○	○	○	○	○	○	○

Kuvittele, että hän on uusi pääministeri. Mikä on valinnan herättämä tunne sinussa? *

Valitse sopivin vaihtoehto:

	1 Erittäin epämiellyttävä	2	3	4 Neutraali	5	6	7 Erittäin miellyttävä
**Valitse sopivin vaihtoehto**	◯	◯	◯	◯	◯	◯	◯

Miten suuri tämän tunteen voimakkuus on? *

Valitse sopivin vaihtoehto:

	1 Erittäin pieni	2	3	4 Kohtalaisen suuri	5	6	7 Erittäin suuri
**Valitse sopivin vaihtoehto**	◯	◯	◯	◯	◯	◯	◯

Part1page5

Suomi vuonna 2020

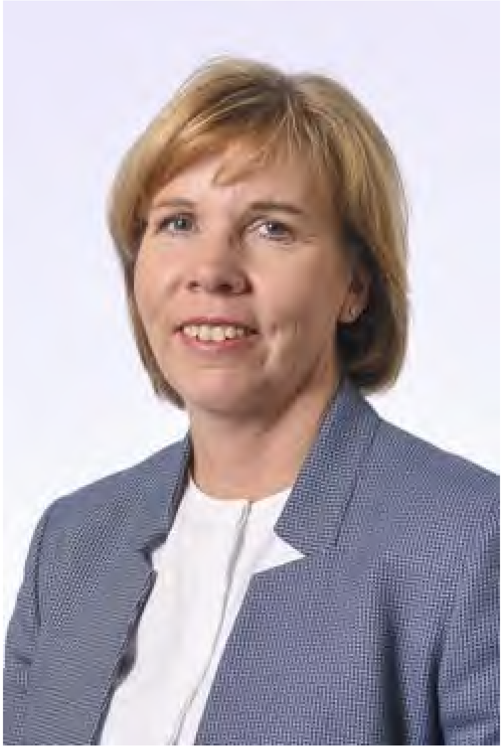

Anna-Maja Henriksson, RKP

Kuinka tuttu tämä poliitikko on sinulle? Arvioi työtä poliitikkona älä yksityishenkilönä. *

Valitse sopivin vaihtoehto:

	1 Ei lainkaan	2	3	4 Jonkin verran	5	6	7 Erittäin tuttu
**Valitse sopivin vaihtoehto**	◯	◯	◯	◯	◯	◯	◯

arvioi työtä poliitikkona, ei yksityiselämää

Vastaa väittämään: Hän on sopiva Suomen pääministeriksi. *

Valitse sopivin vaihtoehto:

	1 Vahvasti eri mieltä	2	3	4 Neutraali	5	6	7 Vahvasti samaa mieltä
**Valitse sopivin vaihtoehto**	○	○	○	○	○	○	○

Arvioi ehdokasta vastaamalla seuraaviin väittämiin: *

Valitse sopivin vaihtoehto:

	1 Vahvasti eri mieltä	2	3	4 Neutraali	5	6	7 Vahvasti samaa mieltä
**Hän on luotettava**	○	○	○	○	○	○	○
**Hän on yhteistyökykyinen**	○	○	○	○	○	○	○
**Hän on oikeudenmukainen**	○	○	○	○	○	○	○
**Hän toimii Suomen hyväksi**	○	○	○	○	○	○	○
**Hän on päätöskykyinen**	○	○	○	○	○	○	○

Kuvittele, että hän on uusi pääministeri. Mikä on valinnan herättämä tunne sinussa? *

Valitse sopivin vaihtoehto:

	1 Erittäin epämiellyttävä	2	3	4 Neutraali	5	6	7 Erittäin miellyttävä
**Valitse sopivin vaihtoehto**	◯	◯	◯	◯	◯	◯	◯

Miten suuri tämän tunteen voimakkuus on? *

Valitse sopivin vaihtoehto:

	1 Erittäin pieni	2	3	4 Kohtalaisen suuri	5	6	7 Erittäin suuri
**Valitse sopivin vaihtoehto**	◯	◯	◯	◯	◯	◯	◯

Part1page6

Suomi vuonna 2020

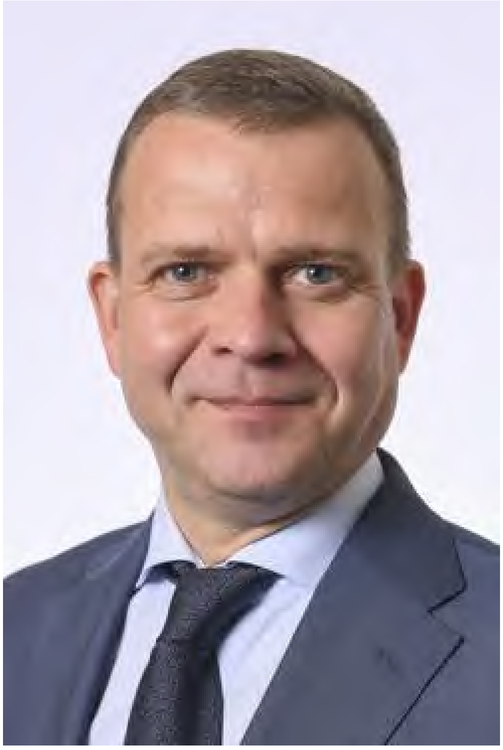

Petteri Orpo, Kok.

Kuinka tuttu tämä poliitikko on sinulle? Arvioi työtä poliitikkona älä yksityishenkilönä. *

Valitse sopivin vaihtoehto:

	1 Ei lainkaan	2	3	4 Jonkin verran	5	6	7 Erittäin tuttu
**Valitse sopivin vaihtoehto**	○	○	○	○	○	○	○

arvioi työtä poliitikkona, ei yksityiselämää

Vastaa väittämään: Hän on sopiva Suomen pääministeriksi. *

Valitse sopivin vaihtoehto:

	1 Vahvasti eri mieltä	2	3	4 Neutraali	5	6	7 Vahvasti samaa mieltä
**Valitse sopivin vaihtoehto**	◯	◯	◯	◯	◯	◯	◯

Arvioi ehdokasta vastaamalla seuraaviin väittämiin: *

Valitse sopivin vaihtoehto:

	1 Vahvasti eri mieltä	2	3	4 Neutraali	5	6	7 Vahvasti samaa mieltä
**Hän on luotettava**	◯	◯	◯	◯	◯	◯	◯
**Hän on yhteistyökykyinen**	◯	◯	◯	◯	◯	◯	◯
**Hän on oikeudenmukainen**	◯	◯	◯	◯	◯	◯	◯
**Hän toimii Suomen hyväksi**	◯	◯	◯	◯	◯	◯	◯
**Hän on päätöskykyinen**	◯	◯	◯	◯	◯	◯	◯

Kuvittele, että hän on uusi pääministeri. Mikä on valinnan herättämä tunne sinussa? *

Valitse sopivin vaihtoehto:

	1 Erittäin epämiellyttävä	2	3	4 Neutraali	5	6	7 Erittäin miellyttävä
**Valitse sopivin vaihtoehto**	◯	◯	◯	◯	◯	◯	◯

Miten suuri tämän tunteen voimakkuus on? *

Valitse sopivin vaihtoehto:

	1 Erittäin pieni	2	3	4 Kohtalaisen suuri	5	6	7 Erittäin suuri
**Valitse sopivin vaihtoehto**	◯	◯	◯	◯	◯	◯	◯

Part1page7

Suomi vuonna 2020

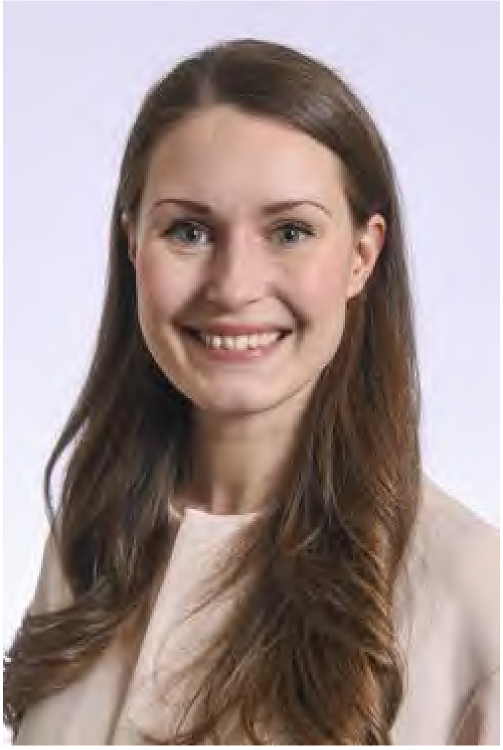

Sanna Marin, SDP

Kuinka tuttu tämä poliitikko on sinulle? Arvioi työtä poliitikkona älä yksityishenkilönä. *

Valitse sopivin vaihtoehto:

	1 Ei lainkaan	2	3	4 Jonkin verran	5	6	7 Erittäin tuttu
**Valitse sopivin vaihtoehto**	◯	◯	◯	◯	◯	◯	◯

arvioi työtä poliitikkona, ei yksityiselämää

Vastaa väittämään: Hän on sopiva Suomen pääministeriksi. *

Valitse sopivin vaihtoehto:

	1 Vahvasti eri mieltä	2	3	4 Neutraali	5	6	7 Vahvasti samaa mieltä
**Valitse sopivin vaihtoehto**	◯	◯	◯	◯	◯	◯	◯

Arvioi ehdokasta vastaamalla seuraaviin väittämiin: *

Valitse sopivin vaihtoehto:

	1 Vahvasti eri mieltä	2	3	4 Neutraali	5	6	7 Vahvasti samaa mieltä
**Hän on luotettava**	◯	◯	◯	◯	◯	◯	◯
**Hän on yhteistyökykyinen**	◯	◯	◯	◯	◯	◯	◯
**Hän on oikeudenmukainen**	◯	◯	◯	◯	◯	◯	◯
**Hän toimii Suomen hyväksi**	◯	◯	◯	◯	◯	◯	◯
**Hän on päätöskykyinen**	◯	◯	◯	◯	◯	◯	◯

Kuvittele, että hän on uusi pääministeri. Mikä on valinnan herättämä tunne sinussa? *

Valitse sopivin vaihtoehto:

	1 Erittäin epämiellyttävä	2	3	4 Neutraali	5	6	7 Erittäin miellyttävä
**Valitse sopivin vaihtoehto**	◯	◯	◯	◯	◯	◯	◯

Miten suuri tämän tunteen voimakkuus on? *

Valitse sopivin vaihtoehto:

	1 Erittäin pieni	2	3	4 Kohtalaisen suuri	5	6	7 Erittäin suuri
**Valitse sopivin vaihtoehto**	◯	◯	◯	◯	◯	◯	◯

Part1page8

Suomi vuonna 2020

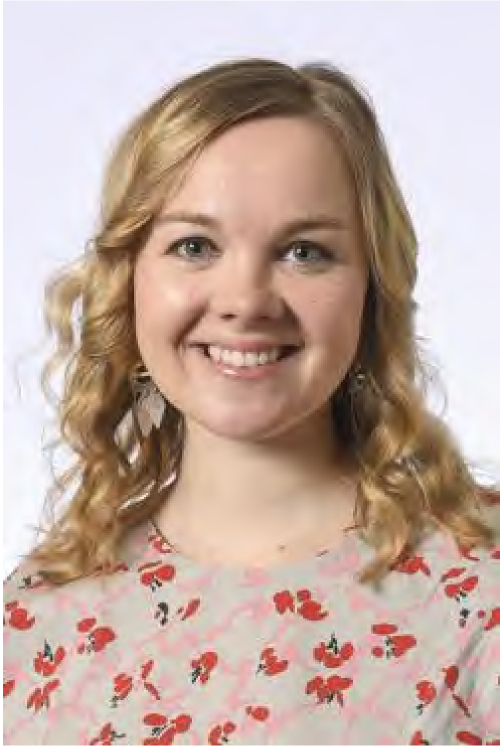

Katri Kulmuni, Kesk.

Kuinka tuttu tämä poliitikko on sinulle? Arvioi työtä poliitikkona älä yksityishenkilönä. *

Valitse sopivin vaihtoehto:

	1 Ei lainkaan	2	3	4 Jonkin verran	5	6	7 Erittäin tuttu
**Valitse sopivin vaihtoehto**	○	○	○	○	○	○	○

arvioi työtä poliitikkona, ei yksityiselämää

Vastaa väittämään: Hän on sopiva Suomen pääministeriksi. *

Valitse sopivin vaihtoehto:

	1 Vahvasti eri mieltä	2	3	4 Neutraali	5	6	7 Vahvasti samaa mieltä
**Valitse sopivin vaihtoehto**	◯	◯	◯	◯	◯	◯	◯

Arvioi ehdokasta vastaamalla seuraaviin väittämiin: *

Valitse sopivin vaihtoehto:

	1 Vahvasti eri mieltä	2	3	4 Neutraali	5	6	7 Vahvasti samaa mieltä
**Hän on luotettava**	◯	◯	◯	◯	◯	◯	◯
**Hän on yhteistyökykyinen**	◯	◯	◯	◯	◯	◯	◯
**Hän on oikeudenmukainen**	◯	◯	◯	◯	◯	◯	◯
**Hän toimii Suomen hyväksi**	◯	◯	◯	◯	◯	◯	◯
**Hän on päätöskykyinen**	◯	◯	◯	◯	◯	◯	◯

Kuvittele, että hän on uusi pääministeri. Mikä on valinnan herättämä tunne sinussa? *

Valitse sopivin vaihtoehto:

	1 Erittäin epämiellyttävä	2	3	4 Neutraali	5	6	7 Erittäin miellyttävä
**Valitse sopivin vaihtoehto**	◯	◯	◯	◯	◯	◯	◯

Miten suuri tämän tunteen voimakkuus on? *

Valitse sopivin vaihtoehto:

	1 Erittäin pieni	2	3	4 Kohtalaisen suuri	5	6	7 Erittäin suuri
**Valitse sopivin vaihtoehto**	◯	◯	◯	◯	◯	◯	◯

Part1page9

Suomi vuonna 2020

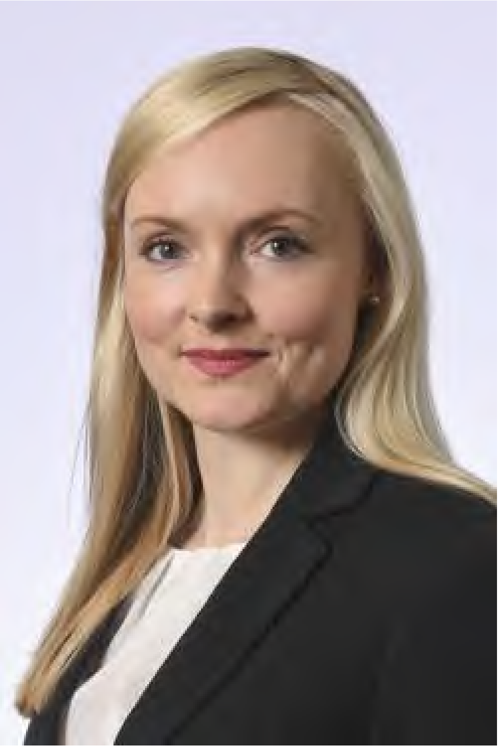

Mari Ohisalo, Vihr.

Kuinka tuttu tämä poliitikko on sinulle? Arvioi työtä poliitikkona älä yksityishenkilönä. *

Valitse sopivin vaihtoehto:

	1 Ei lainkaan	2	3	4 Jonkin verran	5	6	7 Erittäin tuttu
**Valitse sopivin vaihtoehto**	○	○	○	○	○	○	○

arvioi työtä poliitikkona, ei yksityiselämää

Vastaa väittämään: Hän on sopiva Suomen pääministeriksi. *

Valitse sopivin vaihtoehto:

	1 Vahvasti eri mieltä	2	3	4 Neutraali	5	6	7 Vahvasti samaa mieltä
**Valitse sopivin vaihtoehto**	○	○	○	○	○	○	○

Arvioi ehdokasta vastaamalla seuraaviin väittämiin: *

Valitse sopivin vaihtoehto:

	1 Vahvasti eri mieltä	2	3	4 Neutraali	5	6	7 Vahvasti samaa mieltä
**Hän on luotettava**	○	○	○	○	○	○	○
**Hän on yhteistyökykyinen**	○	○	○	○	○	○	○
**Hän on oikeudenmukainen**	○	○	○	○	○	○	○
**Hän toimii Suomen hyväksi**	○	○	○	○	○	○	○
**Hän on päätöskykyinen**	○	○	○	○	○	○	○

Kuvittele, että hän on uusi pääministeri. Mikä on valinnan herättämä tunne sinussa? *

Valitse sopivin vaihtoehto:

	1 Erittäin epämiellyttävä	2	3	4 Neutraali	5	6	7 Erittäin miellyttävä
**Valitse sopivin vaihtoehto**	◯	◯	◯	◯	◯	◯	◯

Miten suuri tämän tunteen voimakkuus on? *

Valitse sopivin vaihtoehto:

	1 Erittäin pieni	2	3	4 Kohtalaisen suuri	5	6	7 Erittäin suuri
**Valitse sopivin vaihtoehto**	◯	◯	◯	◯	◯	◯	◯

Part2realvoting

Vastaa seuraaviin kysymyksiin liittyen äänestämiseesi.

Jos vaalit pidettäisiin nyt, kenen johtamaa (=pääministeriehdokas) puoluetta äänestäisit? Valitse yksi. *

Valitse **vain yksi** seuraavista:

- ◯ 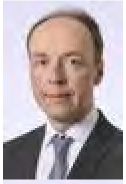 Perussuomalaiset (PS)
- ◯ 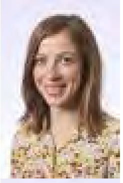 Vasemmistoliitto (Vas.)
- ◯ 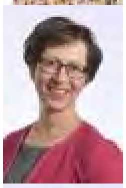 Suomen Kristillisdemokraatit (KD)
- ◯ 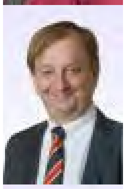 Liike Nyt
- ◯ 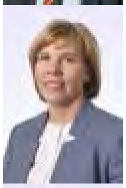 Suomen ruotsalainen kansanpuolue (RKP)
- ◯ 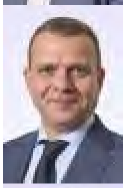 Kansallinen Kokoomus (Kok.)
- ◯ 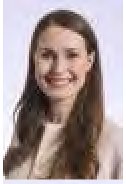 Suomen Sosialidemokraattinen Puolue (SDP)
- ◯ 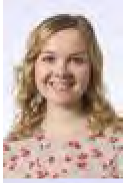 Suomen Keskusta (Kesk.)
- ◯ 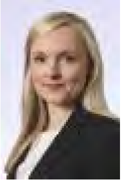 Vihreä liitto (Vihr.)
- ◯ Muu / En halua vastata

Muistele mitä puoluetta äänestit kevään **2019** eduskuntavaaleissa. Valitse yksi vaihtoehto. *

Valitse **vain yksi** seuraavista:

- ◯ Kansallinen Kokoomus (Kok.)
- ◯ Suomen Keskusta (Kesk.)
- ◯ Suomen Sosialidemokraattinen Puolue (SDP)
- ◯ Perussuomalaiset (PS)
- ◯ Vasemmistoliitto (Vas.)
- ◯ Suomen Kristillisdemokraatit (KD)
- ◯ Suomen ruotsalainen kansanpuolue (RKP)
- ◯ Liike Nyt
- ◯ Muu / En halua vastata

Part3scenario

Kuvittele seuraava tilanne Suomeen lähitulevaisuudessa. Lue kuvaus huolellisesti ja ajatuksella.

Kuvittele seuraava tilanne Suomeen vuonna 2025:

Suomen taloudella menee hyvin ja sinun palkasta jää välttämättömien menojen jälkeen reilusti ylimääräistä rahaa. Et ole turvautunut toimeentulotukeen ollenkaan. Tarvitset lääkäriä harvoin ja lääkärissä käynti ei maksa mitään. Työpaikallasi hyödynnetään tekoälyä paljon ja rutiinitöitä on enää vähän. Työaikasi on 6,5 tuntia päivässä ja sinulla on paljon lomia ja vapaa-aikaa. Suomen työttömyys on ennätyksellisen alhaalla. Perustoimeentulo on hyvä työelämän ulkopuolella oleville ihmisille ja sen avulla he voivat opiskella, harrastaa, hoitaa lapsia tai viettää vapaa-aikaa.

Asut talossa ja käytät kulkuneuvoja, joiden päästöt ovat hyvin pienet kehittyneen teknologian vuoksi. Ilmastonmuutos on saatu hyvin hallintaan. Syöt ilmastoa vähän kuormittavaa ruokaa ja se on edullista. Kansainvälisen vakauden seurauksena pakolaisten määrä on vähentynyt ja suurin osa heistä kotiutuu hyvin. Sinä ja muut suomalaiset voitte hyvin ja tulevaisuuden näkymät ovat myönteiset.

Vastaa tähän vain jos seuraavat ehdot täyttyvät:
RandomScene.value (/admin/questions/sa/view/surveyid/245313/gid/216/qid/2017) == 1

Kuvittele seuraava tilanne Suomeen vuonna 2025:

Suomen taloudella menee huonosti ja sinun palkasta ei jää välttämättömien menojen jälkeen ylimääräistä rahaa. Olet turvautunut toimeentulotukeen usein. Tarvitset lääkäriä usein ja lääkärissä käynti maksaa paljon. Työpaikallasi ei hyödynnetä tekoälyä ollenkaan ja rutiinitöitä on vielä paljon. Työaikasi on 9,5 tuntia päivässä ja sinulla on vähän lomia ja vapaa-aikaa. Suomen työttömyys on ennätyksellisen korkealla. Perustoimeentulo on huono työelämän ulkopuolella oleville ihmisille ja sen avulla he eivät voi opiskella, harrastaa, hoitaa lapsia tai viettää vapaa-aikaa.

Asut talossa ja käytät kulkuneuvoja, joiden päästöt ovat hyvin suuret kehittymättömän teknologian vuoksi. Ilmastonmuutosta ei ole saatu hallintaan. Syöt ilmastoa paljon kuormittavaa ruokaa ja se on kallista. Kansainvälisen epävakauden seurauksena pakolaisten määrä on kasvanut ja suurin osa heistä syrjäytyy. Sinä ja muut suomalaiset voitte huonosti ja tulevaisuuden näkymät ovat kielteiset.

Vastaa tähän vain jos seuraavat ehdot täyttyvät:
RandomScene.value (/admin/questions/sa/view/surveyid/245313/gid/216/qid/2017) == 2

Part4page1

Suomi vuonna 2025

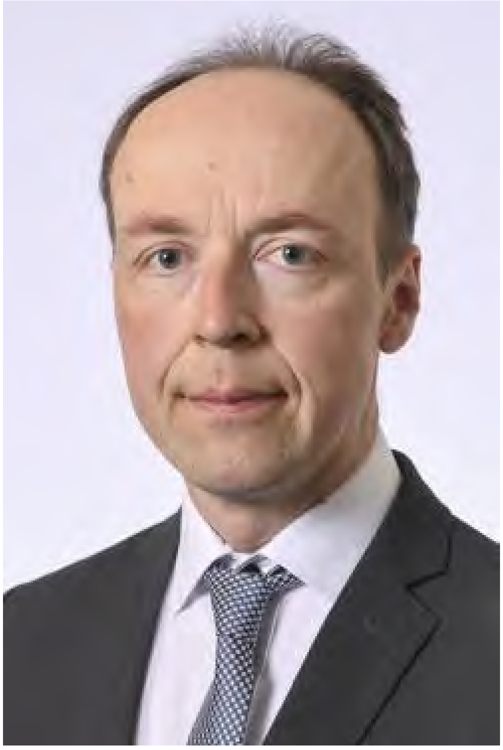

Jussi Halla-aho, PS

Vastaa väittämään: Hän on sopiva Suomen pääministeriksi. *

Valitse sopivin vaihtoehto:

	1 Vahvasti eri mieltä	2	3	4 Neutraali	5	6	7 Vahvasti samaa mieltä
**Valitse sopivin vaihtoehto**	◯	◯	◯	◯	◯	◯	◯

Arvioi ehdokasta vastaamalla seuraaviin väittämiin: *

Valitse sopivin vaihtoehto:

	1 Vahvasti eri mieltä	2	3	4 Neutraali	5	6	7 Vahvasti samaa mieltä
**Hän on luotettava**	○	○	○	○	○	○	○
**Hän on yhteistyökykyinen**	○	○	○	○	○	○	○
**Hän on oikeudenmukainen**	○	○	○	○	○	○	○
**Hän toimii Suomen hyväksi**	○	○	○	○	○	○	○
**Hän on päätöskykyinen**	○	○	○	○	○	○	○

Kuvittele, että hän on uusi pääministeri. Mikä on valinnan herättämä tunne sinussa? *

Valitse sopivin vaihtoehto:

	1 Erittäin epämiellyttävä	2	3	4 Neutraali	5	6	7 Erittäin miellyttävä
**Valitse sopivin vaihtoehto**	○	○	○	○	○	○	○

Miten suuri tämän tunteen voimakkuus on? *

Valitse sopivin vaihtoehto:

	1 Erittäin pieni	2	3	4 Kohtalaisen suuri	5	6	7 Erittäin suuri
**Valitse sopivin vaihtoehto**	◯	◯	◯	◯	◯	◯	◯

Part4page2

Suomi vuonna 2025

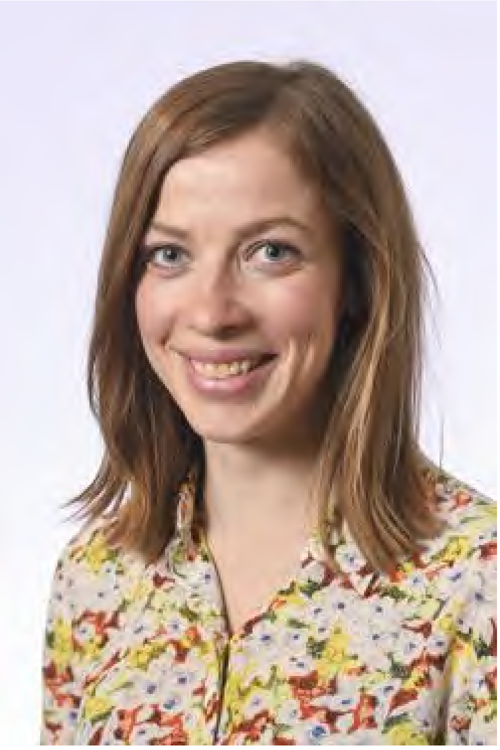

Li Andersson, Vas.

Vastaa väittämään: Hän on sopiva Suomen pääministeriksi. *

Valitse sopivin vaihtoehto:

	1 Vahvasti eri mieltä	2	3	4 Neutraali	5	6	7 Vahvasti samaa mieltä
**Valitse sopivin vaihtoehto**	◯	◯	◯	◯	◯	◯	◯

Arvioi ehdokasta vastaamalla seuraaviin väittämiin: *

Valitse sopivin vaihtoehto:

	1 Vahvasti eri mieltä	2	3	4 Neutraali	5	6	7 Vahvasti samaa mieltä
**Hän on luotettava**	◯	◯	◯	◯	◯	◯	◯
**Hän on yhteistyökykyinen**	◯	◯	◯	◯	◯	◯	◯
**Hän on oikeudenmukainen**	◯	◯	◯	◯	◯	◯	◯
**Hän toimii Suomen hyväksi**	◯	◯	◯	◯	◯	◯	◯
**Hän on päätöskykyinen**	◯	◯	◯	◯	◯	◯	◯

Kuvittele, että hän on uusi pääministeri. Mikä on valinnan herättämä tunne sinussa? *

Valitse sopivin vaihtoehto:

	1 Erittäin epämiellyttävä	2	3	4 Neutraali	5	6	7 Erittäin miellyttävä
**Valitse sopivin vaihtoehto**	◯	◯	◯	◯	◯	◯	◯

Miten suuri tämän tunteen voimakkuus on? *

Valitse sopivin vaihtoehto:

	1 Erittäin pieni	2	3	4 Kohtalaisen suuri	5	6	7 Erittäin suuri
**Valitse sopivin vaihtoehto**	◯	◯	◯	◯	◯	◯	◯

Part4page3

Suomi vuonna 2025

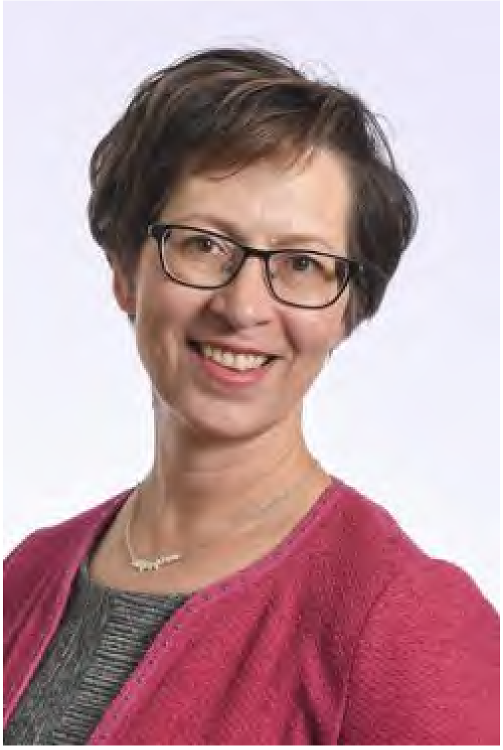

Sari Essayah, KD

Vastaa väittämään: Hän on sopiva Suomen pääministeriksi. *

Valitse sopivin vaihtoehto:

	1 Vahvasti eri mieltä	2	3	4 Neutraali	5	6	7 Vahvasti samaa mieltä
**Valitse sopivin vaihtoehto**	◯	◯	◯	◯	◯	◯	◯

Arvioi ehdokasta vastaamalla seuraaviin väittämiin: *

Valitse sopivin vaihtoehto:

	1 Vahvasti eri mieltä	2	3	4 Neutraali	5	6	7 Vahvasti samaa mieltä
**Hän on luotettava**	○	○	○	○	○	○	○
**Hän on yhteistyökykyinen**	○	○	○	○	○	○	○
**Hän on oikeudenmukainen**	○	○	○	○	○	○	○
**Hän toimii Suomen hyväksi**	○	○	○	○	○	○	○
**Hän on päätöskykyinen**	○	○	○	○	○	○	○

Kuvittele, että hän on uusi pääministeri. Mikä on valinnan herättämä tunne sinussa? *

Valitse sopivin vaihtoehto:

	1 Erittäin epämiellyttävä	2	3	4 Neutraali	5	6	7 Erittäin miellyttävä
**Valitse sopivin vaihtoehto**	○	○	○	○	○	○	○

Miten suuri tämän tunteen voimakkuus on? *

Valitse sopivin vaihtoehto:

	1 Erittäin pieni	2	3	4 Kohtalaisen suuri	5	6	7 Erittäin suuri
**Valitse sopivin vaihtoehto**	◯	◯	◯	◯	◯	◯	◯

Part4page4

Suomi vuonna 2025

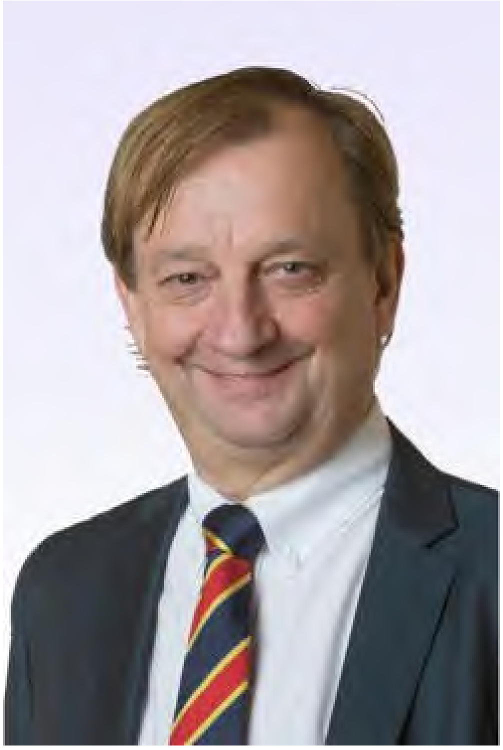

Harry Harkimo, Liike Nyt

Vastaa väittämään: Hän on sopiva Suomen pääministeriksi. *

Valitse sopivin vaihtoehto:

	1 Vahvasti eri mieltä	2	3	4 Neutraali	5	6	7 Vahvasti samaa mieltä
**Valitse sopivin vaihtoehto**	◯	◯	◯	◯	◯	◯	◯

Arvioi ehdokasta vastaamalla seuraaviin väittämiin: *

Valitse sopivin vaihtoehto:

	1 Vahvasti eri mieltä	2	3	4 Neutraali	5	6	7 Vahvasti samaa mieltä
**Hän on luotettava**	◯	◯	◯	◯	◯	◯	◯
**Hän on yhteistyökykyinen**	◯	◯	◯	◯	◯	◯	◯
**Hän on oikeudenmukainen**	◯	◯	◯	◯	◯	◯	◯
**Hän toimii Suomen hyväksi**	◯	◯	◯	◯	◯	◯	◯
**Hän on päätöskykyinen**	◯	◯	◯	◯	◯	◯	◯

Kuvittele, että hän on uusi pääministeri. Mikä on valinnan herättämä tunne sinussa? *

Valitse sopivin vaihtoehto:

	1 Erittäin epämiellyttävä	2	3	4 Neutraali	5	6	7 Erittäin miellyttävä
**Valitse sopivin vaihtoehto**	◯	◯	◯	◯	◯	◯	◯

Miten suuri tämän tunteen voimakkuus on? *

Valitse sopivin vaihtoehto:

	1 Erittäin pieni	2	3	4 Kohtalaisen suuri	5	6	7 Erittäin suuri
**Valitse sopivin vaihtoehto**	◯	◯	◯	◯	◯	◯	◯

Part4page5

Suomi vuonna 2025

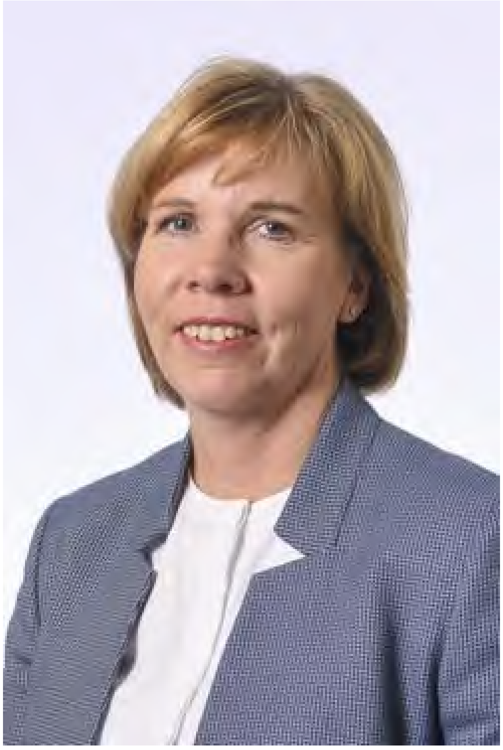

Anna-Maja Henriksson, RKP

Vastaa väittämään: Hän on sopiva Suomen pääministeriksi. *

Valitse sopivin vaihtoehto:

	1 Vahvasti eri mieltä	2	3	4 Neutraali	5	6	7 Vahvasti samaa mieltä
**Valitse sopivin vaihtoehto**	◯	◯	◯	◯	◯	◯	◯

Arvioi ehdokasta vastaamalla seuraaviin väittämiin: *

Valitse sopivin vaihtoehto:

	1 Vahvasti eri mieltä	2	3	4 Neutraali	5	6	7 Vahvasti samaa mieltä
**Hän on luotettava**	◯	◯	◯	◯	◯	◯	◯
**Hän on yhteistyökykyinen**	◯	◯	◯	◯	◯	◯	◯
**Hän on oikeudenmukainen**	◯	◯	◯	◯	◯	◯	◯
**Hän toimii Suomen hyväksi**	◯	◯	◯	◯	◯	◯	◯
**Hän on päätöskykyinen**	◯	◯	◯	◯	◯	◯	◯

Kuvittele, että hän on uusi pääministeri. Mikä on valinnan herättämä tunne sinussa? *

Valitse sopivin vaihtoehto:

	1 Erittäin epämiellyttävä	2	3	4 Neutraali	5	6	7 Erittäin miellyttävä
**Valitse sopivin vaihtoehto**	◯	◯	◯	◯	◯	◯	◯

Miten suuri tämän tunteen voimakkuus on? *

Valitse sopivin vaihtoehto:

	1 Erittäin pieni	2	3	4 Kohtalaisen suuri	5	6	7 Erittäin suuri
**Valitse sopivin vaihtoehto**	◯	◯	◯	◯	◯	◯	◯

Part4page6

Suomi vuonna 2025

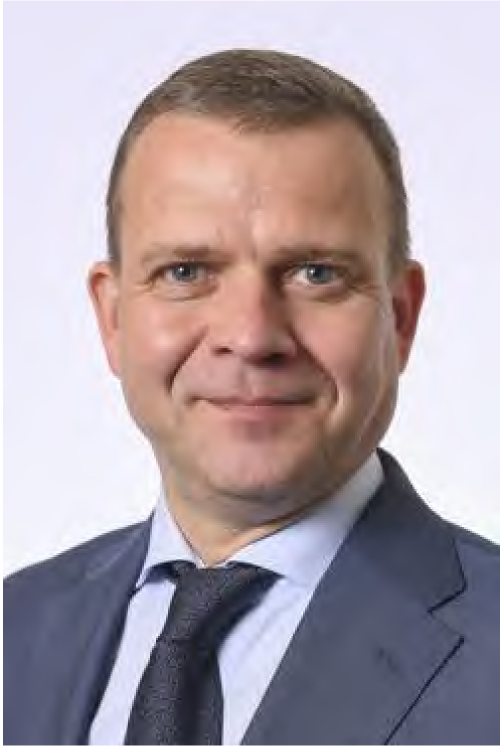

Petteri Orpo, Kok.

Vastaa väittämään: Hän on sopiva Suomen pääministeriksi. *

Valitse sopivin vaihtoehto:

	1 Vahvasti eri mieltä	2	3	4 Neutraali	5	6	7 Vahvasti samaa mieltä
**Valitse sopivin vaihtoehto**	◯	◯	◯	◯	◯	◯	◯

Arvioi ehdokasta vastaamalla seuraaviin väittämiin: *

Valitse sopivin vaihtoehto:

	1 Vahvasti eri mieltä	2	3	4 Neutraali	5	6	7 Vahvasti samaa mieltä
**Hän on luotettava**	◯	◯	◯	◯	◯	◯	◯
**Hän on yhteistyökykyinen**	◯	◯	◯	◯	◯	◯	◯
**Hän on oikeudenmukainen**	◯	◯	◯	◯	◯	◯	◯
**Hän toimii Suomen hyväksi**	◯	◯	◯	◯	◯	◯	◯
**Hän on päätöskykyinen**	◯	◯	◯	◯	◯	◯	◯

Kuvittele, että hän on uusi pääministeri. Mikä on valinnan herättämä tunne sinussa? *

Valitse sopivin vaihtoehto:

	1 Erittäin epämiellyttävä	2	3	4 Neutraali	5	6	7 Erittäin miellyttävä
**Valitse sopivin vaihtoehto**	◯	◯	◯	◯	◯	◯	◯

Miten suuri tämän tunteen voimakkuus on? *

Valitse sopivin vaihtoehto:

	1 Erittäin pieni	2	3	4 Kohtalaisen suuri	5	6	7 Erittäin suuri
**Valitse sopivin vaihtoehto**	◯	◯	◯	◯	◯	◯	◯

Part4page7

Suomi vuonna 2025

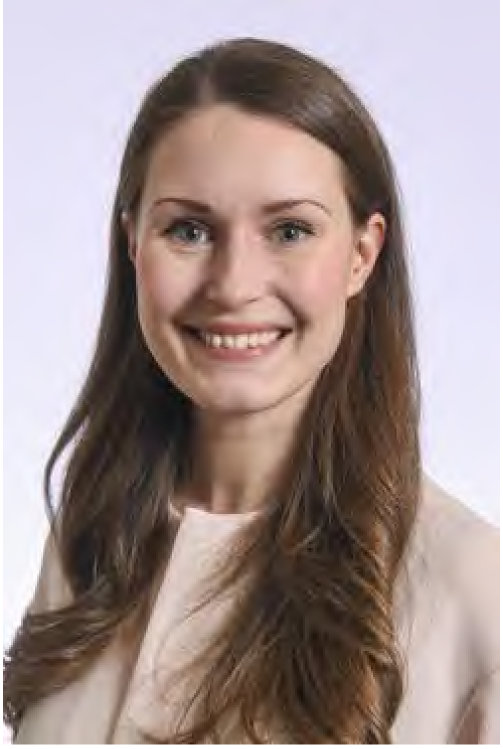

Sanna Marin, SDP

Vastaa väittämään: Hän on sopiva Suomen pääministeriksi. *

Valitse sopivin vaihtoehto:

	1 Vahvasti eri mieltä	2	3	4 Neutraali	5	6	7 Vahvasti samaa mieltä
**Valitse sopivin vaihtoehto**	◯	◯	◯	◯	◯	◯	◯

Arvioi ehdokasta vastaamalla seuraaviin väittämiin: *

Valitse sopivin vaihtoehto:

	1 Vahvasti eri mieltä	2	3	4 Neutraali	5	6	7 Vahvasti samaa mieltä
**Hän on luotettava**	◯	◯	◯	◯	◯	◯	◯
**Hän on yhteistyökykyinen**	◯	◯	◯	◯	◯	◯	◯
**Hän on oikeudenmukainen**	◯	◯	◯	◯	◯	◯	◯
**Hän toimii Suomen hyväksi**	◯	◯	◯	◯	◯	◯	◯
**Hän on päätöskykyinen**	◯	◯	◯	◯	◯	◯	◯

Kuvittele, että hän on uusi pääministeri. Mikä on valinnan herättämä tunne sinussa? *

Valitse sopivin vaihtoehto:

	1 Erittäin epämiellyttävä	2	3	4 Neutraali	5	6	7 Erittäin miellyttävä
**Valitse sopivin vaihtoehto**	◯	◯	◯	◯	◯	◯	◯

Miten suuri tämän tunteen voimakkuus on? *

Valitse sopivin vaihtoehto:

	1 Erittäin pieni	2	3	4 Kohtalaisen suuri	5	6	7 Erittäin suuri
**Valitse sopivin vaihtoehto**	○	○	○	○	○	○	○

Part4page8

Suomi vuonna 2025

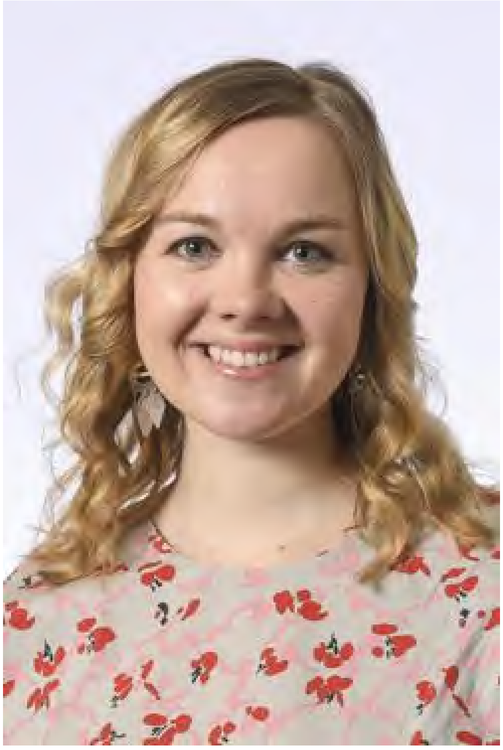

Katri Kulmuni, Kesk.

Vastaa väittämään: Hän on sopiva Suomen pääministeriksi. *

Valitse sopivin vaihtoehto:

	1 Vahvasti eri mieltä	2	3	4 Neutraali	5	6	7 Vahvasti samaa mieltä
**Valitse sopivin vaihtoehto**	◯	◯	◯	◯	◯	◯	◯

Arvioi ehdokasta vastaamalla seuraaviin väittämiin: *

Valitse sopivin vaihtoehto:

	1 Vahvasti eri mieltä	2	3	4 Neutraali	5	6	7 Vahvasti samaa mieltä
**Hän on luotettava**	◯	◯	◯	◯	◯	◯	◯
**Hän on yhteistyökykyinen**	◯	◯	◯	◯	◯	◯	◯
**Hän on oikeudenmukainen**	◯	◯	◯	◯	◯	◯	◯
**Hän toimii Suomen hyväksi**	◯	◯	◯	◯	◯	◯	◯
**Hän on päätöskykyinen**	◯	◯	◯	◯	◯	◯	◯

Kuvittele, että hän on uusi pääministeri. Mikä on valinnan herättämä tunne sinussa? *

Valitse sopivin vaihtoehto:

	1 Erittäin epämiellyttävä	2	3	4 Neutraali	5	6	7 Erittäin miellyttävä
**Valitse sopivin vaihtoehto**	◯	◯	◯	◯	◯	◯	◯

Miten suuri tämän tunteen voimakkuus on? *

Valitse sopivin vaihtoehto:

	1 Erittäin pieni	2	3	4 Kohtalaisen suuri	5	6	7 Erittäin suuri
**Valitse sopivin vaihtoehto**	◯	◯	◯	◯	◯	◯	◯

Part4page9

Suomi vuonna 2025

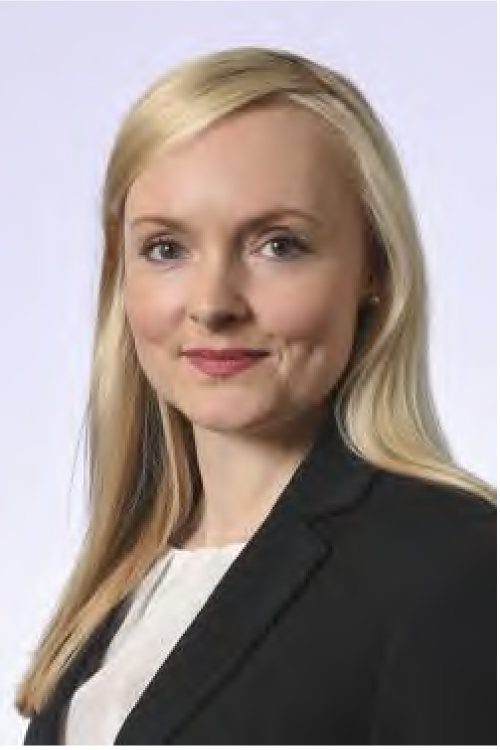

Mari Ohisalo, Vihr.

Vastaa väittämään: Hän on sopiva Suomen pääministeriksi. *

Valitse sopivin vaihtoehto:

	1 Vahvasti eri mieltä	2	3	4 Neutraali	5	6	7 Vahvasti samaa mieltä
**Valitse sopivin vaihtoehto**	◯	◯	◯	◯	◯	◯	◯

Arvioi ehdokasta vastaamalla seuraaviin väittämiin: *

Valitse sopivin vaihtoehto:

	1 Vahvasti eri mieltä	2	3	4 Neutraali	5	6	7 Vahvasti samaa mieltä
**Hän on luotettava**	◯	◯	◯	◯	◯	◯	◯
**Hän on yhteistyökykyinen**	◯	◯	◯	◯	◯	◯	◯
**Hän on oikeudenmukainen**	◯	◯	◯	◯	◯	◯	◯
**Hän toimii Suomen hyväksi**	◯	◯	◯	◯	◯	◯	◯
**Hän on päätöskykyinen**	◯	◯	◯	◯	◯	◯	◯

Kuvittele, että hän on uusi pääministeri. Mikä on valinnan herättämä tunne sinussa? *

Valitse sopivin vaihtoehto:

	1 Erittäin epämiellyttävä	2	3	4 Neutraali	5	6	7 Erittäin miellyttävä
**Valitse sopivin vaihtoehto**	◯	◯	◯	◯	◯	◯	◯

Miten suuri tämän tunteen voimakkuus on? *

Valitse sopivin vaihtoehto:

	1 Erittäin pieni	2	3	4 Kohtalaisen suuri	5	6	7 Erittäin suuri
**Valitse sopivin vaihtoehto**	◯	◯	◯	◯	◯	◯	◯

FinalPage

Kysely on päättynyt, kiitämme sinua lämpimästi osallistumisestasi tutkimukseemme!

Mikäli annoit email-osoitteen ja voitat arvonnassa, otamme sinuun yhteyttä.

Jos haluat antaa palautetta liittyen kyselyyn tai tutkimukseen, kirjoita ne alle.

Vastauksesi:

Lähetä vastaukset.
Kiitos vastauksistasi!
