# Supplementary material for: Behavioral science of voting in hypothetical utopia and dystopia scenarios: a predictive modeling approach
Source: Front Psychol. 2026 Jan 12;16:1713314. doi: 10.3389/fpsyg.2025.1713314 (PMC12833430; doi:10.3389/fpsyg.2025.1713314)
Supplement: Supplementary file 3 [file Data_Sheet_3.docx]

**Supplementary Materials**

**Content**

[Table S1. Performance for the test data for the boosting model. 2](#_Toc208474519)

[Figure S1. Relative importance of feature interactions in the boosting model. 2](#_Toc208474520)

[Figure S2. Relative politician valuations. 3](#_Toc208474521)

[Overview of machine learning in political science research 3](#_Toc208474522)

[Details of the Bayesian linear regression 4](#_Toc208474523)

[References 5](#_Toc208474524)

Table S1. Performance for the test data for the boosting model. Table contains R^2^ values for each response and politician and their pair-wise combinations for test data. Number of subjects were n=998 (training) and n=111 (testing).

|  | **Politician** | | | | | | | | |  |
| --- | --- | --- | --- | --- | --- | --- | --- | --- | --- | --- |
|  | AH | HH | JH | KK | LA | MO | PO | SM | SE | **All** |
| Suitability | 0.10 | 0.07 | 0.53 | 0.17 | 0.42 | 0.45 | 0.34 | 0.38 | 0.06 | **0.39** |
| Trustworthiness | 0.10 | 0.09 | 0.45 | 0.19 | 0.34 | 0.37 | 0.29 | 0.32 | 0.04 | **0.33** |
| Cooperationskill | 0.13 | 0.08 | 0.44 | 0.10 | 0.28 | 0.36 | 0.28 | 0.25 | 0.02 | **0.32** |
| Righteousness | 0.18 | 0.09 | 0.54 | 0.17 | 0.36 | 0.41 | 0.32 | 0.28 | 0.07 | **0.36** |
| Patriotism | 0.12 | 0.07 | 0.43 | 0.06 | 0.35 | 0.42 | 0.21 | 0.30 | 0.03 | **0.28** |
| Decisionmaking | 0.08 | 0.08 | 0.17 | 0.16 | 0.22 | 0.28 | 0.09 | 0.21 | 0.05 | **0.21** |
| **All** | **0.20** | **0.25** | **0.49** | **0.19** | **0.36** | **0.39** | **0.31** | **0.30** | **0.15** | **0.35** |


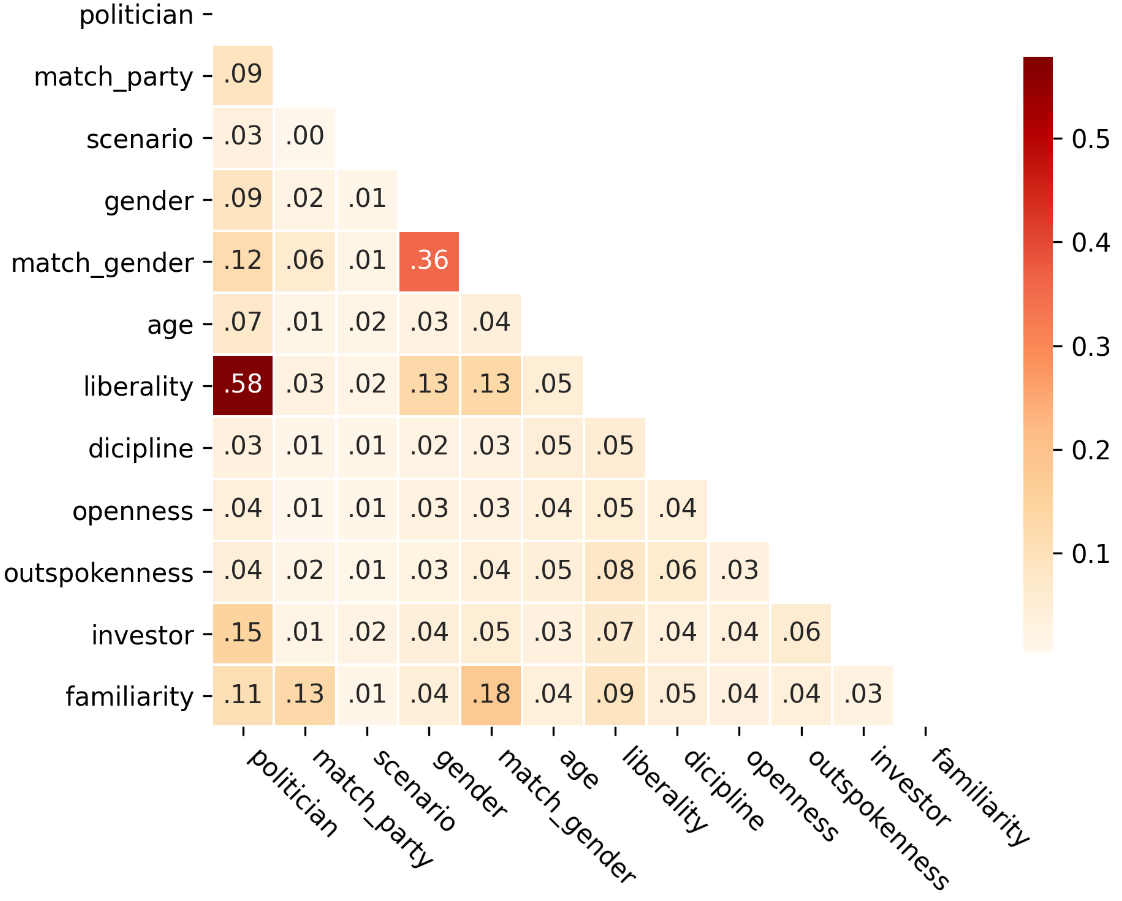


Figure S1. Relative importance of feature interactions in the boosting model. Feature-pair importance’s were computed as pairwise SHAP values by taking absolute values and mean. Here all six responses were summed together, hence each cell represents a summary of 119772 values.

First, we look at the relative popularities of politicians using the estimated random-effects intercepts. The intercepts are depicted in Figure 6 with their 95% HDI estimates. The results were like those obtained for the boosting model (see Fig. 3) with the largest difference being with HH who was ranked 3^rd^ here instead of 8^th^, i.e., the difference in rank being 5 steps.


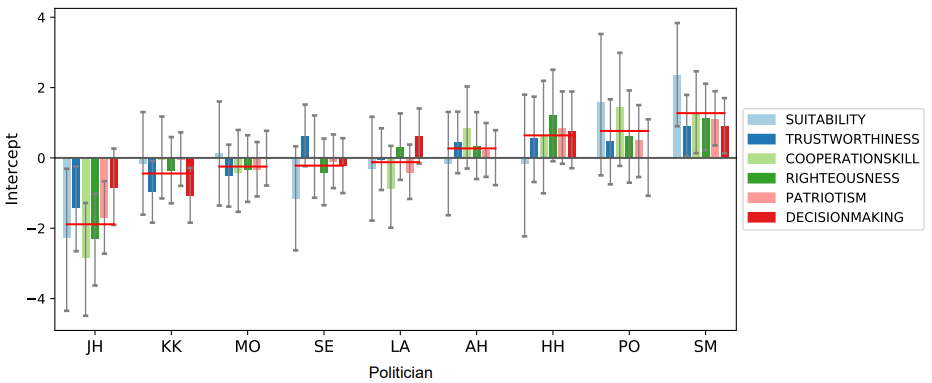


Figure S2. Relative politician valuations. Bars depict random-effects intercepts for politicians in Bayesian linear regression model (including all politicians) that treated politicians as random effects. Gray bars depict HDI at 95%.

Overview of machine learning in political science research

The most common approach in analyzing quantitative survey data in general, and particularly in political science, are classical statistical methods, such as linear regression, cross-tabulation, and structural equation modeling (1). However, methods from the machine learning community are becoming more common, which could be at least partially attributed to the rise of “generalist” models such as Large Language Models (LLMs), e.g., ChatGPT. Predictive modelling is particularly useful in studying political processes where controlled experimentation - e.g., priming or varying context during actual voting - is not feasible (2). The availability of new algorithms, off-the-shelf libraries, and computational resources has expanded the scope of analysis to include both structured tabular data and unstructured data sources, such as text and images. Predictive models have been used to anticipate political behaviors - such as voting preferences inferred from Facebook likes (3), political ideology extracted from Twitter (4), and Brexit voting patterns (5). More recently, supervised ensemble methods have been used to estimate swing voter likelihood with high precision (6), and machine learning paired with natural language processing has been applied to uncover latent voting patterns in the European Parliament (7).

Researchers in political science are increasingly exploring richer data modalities beyond tabular inputs. Graph neural networks have been used to model political polarization by jointly analyzing social media content, user attributes, and online network structures (8). Recent work has emphasized the importance of aligning multimodal inputs - such as speech and visuals from campaign ads or parliamentary sessions - for improved inference (9). Recent work has demonstrated that LLMs can be used to estimate individual-level voting behavior by classifying likely voter turnout and preferences from textual prompts, showing promising alignment with traditional survey-based measures (10). These advancements suggest that predictive political modeling is moving rapidly beyond the constraints of survey-based approaches, integrating increasingly diverse data sources and methods.

Machine learning methods allow fitting complex, non-linear models that maximize the predictive power of the model with new data. In particular, tree-based methods (e.g., CatBoost, XGBoost and Random Forests) are particularly valuable in settings where one wishes to make accurate predictions in the context of a generally unknown data generating process with potential nonlinearities, interactions, and many (potentially irrelevant) covariates (11). For example, with tabular data, we train a model with a large dataset and use it to make predictions of new, unseen data. The objects of interest are the variables rather than the parameters and all available data is used to produce the best possible predictions of the outcome variable(s) (2). Increased model complexity comes with the price of inference and transparency as many machine learning models are “black box” -type which makes it difficult to assess the total effect and associated uncertainty of individual variables in the data. To solve this problem, researchers have developed methods, such as SHAP (12), to make model-agnostic interpretations of model predictions. SHAP can produce estimates for the contribution of individual features towards a specific prediction by generating perturbations of a given instance in the data and observing the effect of these perturbations on the output.

Details of the Bayesian linear regression

Analysis closely follows the steps described in earlier research (13,14) and works related to brms library (15,16). In Bayesian regression, the posterior distributions of model parameters were estimated using the Markov chain Monte Carlo method with the brms R package brms, utilizing in the RStan package in the background ([https://mc-stan.org/rstan](https://mc-stan.org/rstan/)). During the development of the models, we used our best judgment and knowledge of the data to write a few dozen suitable model formulas, fitted those models, and compared them against each other using an approximate leave-one-out cross-validation (LOO) method implemented in R (<https://mc-stan.org/loo>). LOO method ranks models using their fitted posterior probabilities (17), making it easy to pick the models that fit the data well. All our models included 6 outputs which were fitted at the same time and applied a cumulative ordinal distribution with a logit link. We used very weakly informative zero-mean normal priors for regression coefficients (SD 15); otherwise, priors were kept as built-in defaults of brms. The weak prior was chosen according to recommendations to improve convergence and model identifiability, as well as to reduce the risk of overfitting (see <https://github.com/stan-dev/stan/wiki/Prior-Choice-Recommendations>).

In model fitting, we constructed 10 chains of 3000 steps, including 900-step warm-up periods; thus, a total of 10x(3000-900)=21,000 steps were retained to estimate posterior distributions for each model. Convergence of the chains was verified by visual inspection of prediction distributions (posterior predictive check) by comparing the observed data to simulated data from the posterior predictive distribution. The checks demonstrated that our chosen models adequately captured the underlying data structure. We also ensured that the potential scale reduction factor R on split chains was below 1.01. After model fitting, we computed estimates of marginal means of the posterior distributions of terms of interest with their two-tailed posterior probabilities against zero as 2 × min [P (x > 0), P (x < 0)]. For estimating marginal means, we used the *emmeans* (<https://github.com/rvlenth/emmeans>) package for R.

References

1. McNabb DE. Research methods for political science: Quantitative and qualitative methods: Second edition. Research Methods for Political Science: Quantitative and Qualitative Methods: Second Edition. 2015. 1–426 p.

2. Cranmer SJ, Desmarais BA. What can we learn from predictive modeling? Polit Anal. 2017;25(2):145–66.

3. Kristensen JB, Albrechtsen T, Dahl-Nielsen E, Jensen M, Skovrind M, Bornakke T. Parsimonious data: How a single Facebook like predicts voting behavior in multiparty systems. Braunstein LA, editor. PLoS One [Internet]. 2017 Sep 20;12(9):e0184562. Available from: https://dx.plos.org/10.1371/journal.pone.0184562

4. Preotiuc-Pietro D, Hopkins DJ, Liu Y, Ungar L. Beyond binary labels: Political ideology prediction of twitter users. In: ACL 2017 - 55th Annual Meeting of the Association for Computational Linguistics, Proceedings of the Conference (Long Papers). 2017. p. 729–40.

5. Becker SO, Fetzer T, Novy D. Who voted for Brexit? A comprehensive district-level analysis. Econ Policy. 2017;32(92):601–50.

6. Hare C, Kutsuris M. Measuring Swing Voters with a Supervised Machine Learning Ensemble. Polit Anal [Internet]. 2023 Oct 17;31(4):537–53. Available from: https://www.cambridge.org/core/product/identifier/S1047198722000249/type/journal_article

7. Guadarrama Rios M, Zamberlan F, Mavromoustakos Blom P, Rankovic N. Knowing our choices: unveiling true voting patterns through machine learning (ML) and natural language processing (NLP) in European Parliament. Soc Netw Anal Min [Internet]. 2025 Mar 20;15(1):24. Available from: https://link.springer.com/10.1007/s13278-025-01452-9

8. Lyu H, Luo J. Understanding Political Polarization via Jointly Modeling Users, Connections and Multimodal Contents on Heterogeneous Graphs. In: Proceedings of the 30th ACM International Conference on Multimedia [Internet]. New York, NY, USA: ACM; 2022. p. 4072–82. Available from: https://dl.acm.org/doi/10.1145/3503161.3547898

9. Arnold C, Küpfer A. How Alignment Helps Make the Most of Multimodal Data. 2024; Available from: http://arxiv.org/abs/2405.08454

10. von der Heyde L, Haensch AC, Wenz A. Vox Populi, Vox AI? Using Large Language Models to Estimate German Vote Choice. Soc Sci Comput Rev [Internet]. 2025 Apr 26; Available from: https://journals.sagepub.com/doi/10.1177/08944393251337014

11. Montgomery JM, Olivella S. Tree-Based Models for Political Science Data. Am J Pol Sci. 2018;62(3):729–44.

12. Lundberg SM, Lee SI. A unified approach to interpreting model predictions. In: Guyon I, Luxburg U ~V., Bengio S, Wallach H, Fergus R, Vishwanathan S, et al., editors. Advances in Neural Information Processing Systems [Internet]. Curran Associates, Inc.; 2017. p. 4766–75. Available from: http://papers.nips.cc/paper/7062-a-unified-approach-to-interpreting-model-predictions.pdf

13. Alamäki A, Khan UA, Kauttonen J, Schlögl S. An Experiment of AI-Based Assessment: Perspectives of Learning Preferences, Benefits, Intention, Technology Affinity, and Trust. Educ Sci [Internet]. 2024 Dec 17;14(12):1386. Available from: https://www.mdpi.com/2227-7102/14/12/1386

14. V Alamäki A, Mäki M, Kauttonen J. How Students’ Information Sensitivity, Privacy Trade-Offs, and Stages of Customer Journey Affect Consent to Utilize Personal Data. Interdiscip J Information, Knowledge, Manag [Internet]. 2023;18:127–47. Available from: https://www.informingscience.org/Publications/5098

15. Bürkner PC, Vuorre M. Ordinal Regression Models in Psychology: A Tutorial. Adv Methods Pract Psychol Sci. 2019;2(1):77–101.

16. Bürkner PC. Bayesian Item Response Modeling in R with brms and Stan. J Stat Softw. 2021;100(5).

17. Vehtari A, Gelman A, Gabry J. Practical Bayesian model evaluation using leave-one-out cross-validation and WAIC. Stat Comput [Internet]. 2017 Sep 30;27(5):1413–32. Available from: http://link.springer.com/10.1007/s11222-016-9696-4
